# *Cited1* Deficiency Suppresses Intestinal Tumorigenesis

**DOI:** 10.1371/journal.pgen.1003638

**Published:** 2013-08-01

**Authors:** Valérie Méniel, Fei Song, Toby Phesse, Madeleine Young, Oliver Poetz, Lee Parry, John R. Jenkins, Geraint T. Williams, Sally L. Dunwoodie, Alastair Watson, Alan R. Clarke

**Affiliations:** 1 School of Biological Sciences, Cardiff University, Cardiff, Wales, United Kingdom; 2 Department of Gastroenterology, Institute of Translational Medicine, The Henry Wellcome Laboratory, University of Liverpool, England, United Kingdom; 3 Institute of Physiology, Justus-Liebig University Giessen, Giessen, Germany; 4 Cell Signaling and Cell Death, Walter and Eliza Hall Institute for Medical Research, Melbourne, Victoria, Australia; 5 Natural and Medical Sciences Institute at the University of Tuebingen, Reutlingen, Germany; 6 School of Medicine, Cardiff University, Heath Park, Cardiff, Wales, United Kingdom; 7 Developmental and Stem Cell Biology Division, Victor Chang Cardiac Research Institute, Darlinghurst, Sydney, New South Wales, Australia; 8 Faculty of Medicine, University of New South Wales, Kensington, Sydney, New South Wales, Australia; 9 Norwich Medical School, University of East Anglia, Norwich Research Park, Norwich, United Kingdom; Fred Hutchinson Cancer Research Center, United States of America

## Abstract

Conditional deletion of Apc in the murine intestine alters crypt-villus architecture and function. This process is accompanied by multiple changes in gene expression, including upregulation of *Cited1*, whose role in colorectal carcinogenesis is unknown. Here we explore the relevance of Cited1 to intestinal tumorigenesis. We crossed *Cited1* null mice with *Apc^Min/+^* and *AhCre^+^Apc^fl/fl^* mice and determined the impact of *Cited1* deficiency on tumour growth/initiation including tumour multiplicity, cell proliferation, apoptosis and the transcriptome. We show that *Cited1* is up-regulated in both human and murine tumours, and that constitutive deficiency of *Cited1* increases survival in *Apc^Min/+^* mice from 230.5 to 515 days. However, paradoxically, *Cited1* deficiency accentuated nearly all aspects of the immediate phenotype 4 days after conditional deletion of Apc, including an increase in cell death and enhanced perturbation of differentiation, including of the stem cell compartment. Transcriptome analysis revealed multiple pathway changes, including p53, PI3K and Wnt. The activation of Wnt through *Cited1* deficiency correlated with increased transcription of β-catenin and increased levels of dephosphorylated β-catenin. Hence, immediately following deletion of Apc, *Cited1* normally restrains the Wnt pathway at the level of β-catenin. Thus deficiency of *Cited1* leads to hyper-activation of Wnt signaling and an exaggerated Wnt phenotype including elevated cell death. *Cited1* deficiency decreases intestinal tumourigenesis in *Apc^Min/+^* mice and impacts upon a number of oncogenic signaling pathways, including Wnt. This restraint imposed by *Cited1* is consistent with a requirement for *Cited1* to constrain Wnt activity to a level commensurate with optimal adenoma formation and maintenance, and provides one mechanism for tumour repression in the absence of *Cited1*.

## Author Summary

Colorectal cancer is the fourth leading cause of cancer related deaths worldwide, and a key genetic change associated with this disease is mutation of the gene APC. APC encodes a protein which plays a regulatory role in the Wnt signalling pathway. To better understand the mechanisms leading to colorectal cancer after APC loss, we have used a mouse model in which we deleted Apc in the bowel and which developed several characteristics of early stage cancers. Here, we show that after Apc loss, the expression of another gene, Cited1, is increased in mice and human colorectal tumours. To study the role of Cited1 in bowel cancer after loss of Apc, we generated mice mutant for Apc (Min) or mutant for Apc and Cited1 (MinCited1). We observed that MinCited1 mice developed fewer intestinal tumours and lived longer than Min mice suggesting that Cited1 is pro-tumourigenic. However, we also observed that Cited1 deficiency actually increased many of the aspects associated with loss of Apc, including deregulation of the Wnt pathway and cell death. To explain this apparent paradox, we propose a model whereby loss of Cited1, in the context of deregulated Wnt signalling, ‘over-stimulates’ the Wnt pathway, the net effect of which is to inhibit tumourigenesis.

## Introduction

Inactivation of the APC (adenomatous polyposis coli) gene marks one of the earliest events in colorectal tumourigenesis [1], an observation that has given rise to the concept of Apc as a ‘cellular gatekeeper’ protecting against tumourigenesis [2]. This role in suppressing tumour formation has been closely associated with its ability to regulate the level of β-catenin within cells. Thus, Apc normally forms part of the scaffold of proteins that phosphorylate β-catenin and target it for degradation. In the absence of Apc, β-catenin levels become elevated and translocates to the nucleus, where it drives increased transcription of Wnt target genes associated with cell proliferation and cell death [3].

To investigate the biological consequences of *Apc* loss and Wnt activation, we and others have previously used a conditional model of *Apc* loss. In this model, deletion of *Apc* is achieved through use of an inducible *AhCre* transgene, which is responsive to exposure to the xenobiotic β-napthoflavone. Following Cre induction and loss of function of Apc, we observe a range of rapid phenotypic changes. These include promiscuous entry of cells into S phase, loss of differentiated cell types, loss of cell polarity and disorganisation of the crypt-villus structure to the point that discrete crypts are no longer discernable. Apc deficiency also reduces the normal migration of cells along the crypt villus axis, leading to the preferential retention of Apc deficient cells. These changes may all be considered pro-tumourigenic, however we also observe a considerable stress signal within Apc deficient cells, most clearly shown by a significant elevation in apoptosis. These phenotypic changes are accompanied by the expected elevation in levels of nuclear β-catenin and marked changes in the transcriptome [3].

One of the changes we observe in the intestinal epithelial cells of *AhCre^+^Apc^fl/fl^* mice is a strong induction of *Cited1*, a bi-functional transcriptional cofactor which is able to activate or repress transcription in association with other transcription factors [4], [5]. We also found this induction to be dependent upon functional c-Myc as *Cited1* expression returns to basal levels in the additional absence of c-Myc, which completely rescues the phenotype of Apc deficiency [6]. These observations suggest that elevation of *Cited1* may be directly associated with the preneoplastic phenotype.

*Cited1* was originally identified in a mouse melanoma cell line [7]. During vertebrate development, *Cited1* is expressed in progenitors of the heart, limb, axial skeleton, kidney, and placenta [8], [9]. It is implicated as a key co-ordinator during renal epithelial morphogenesis [5] and is involved in mammary gland development [10]. Cited1 is required for placental development with effect on embryo growth and survival [9]. However, Cited1 null mice that survive the early postnatal period are otherwise grossly phenotypically normal [9]. Cited1 is also able to enhance TGF-β signaling and inhibit Wnt signaling depending on cellular context [5], [11]. Both activation and inhibition of transcription are dependent on the CBP/p300 binding C-terminal transcription activation domain CR2, which is conserved throughout the Cited family [5], [11]–[13].

Deregulation of *CITED1* has been implicated in several human cancers, including melanomas, Wilm's tumours and nephroblastomas [7], [14]–[16]. In the mouse, *Cited1* is up-regulated in MMTV-Cre/FloxNeoNeuNT mammary tumours and associates with the transcription factor EGR2 to regulate the expression of the oncogene ErbB2 (HER2, Neu) [17]. Recently it has been shown that Cited1 expression, together with another transcription regulator Six2, specify self-renewing nephron progenitor cells in kidney development and it is suggested that Cited proteins may contribute to the maintenance of the self-renewing capping mesenchyme in the developing kidney [18]–[20]. Thus, although a body of studies have implicated *Cited1* in both embryogenesis and carcinogenesis, its potential role in Wnt-induced intestinal tumourigenesis remains unresolved.

Given the data implicating *Cited1* as a regulator of the Wnt pathway, we have tested the hypothesis that *Cited1* plays a key role in intestinal tumourigenesis. We show that *CITED1* is upregulated in human colorectal cancers and that *Cited1* deficiency increases the survival of *Apc^Min/+^* mice. When crossed into our acute model of *Apc* deficiency, we show that loss of *Cited1* accentuates nearly all aspects of the *Apc* deficient phenotype, including the transcription of a range of oncogenic signaling pathways, including the Wnt pathway.

## Results

### *Cited1/CITED1* is up-regulated in the intestine of Apc deficient mouse models and human colorectal tumours

**Figure 1:**
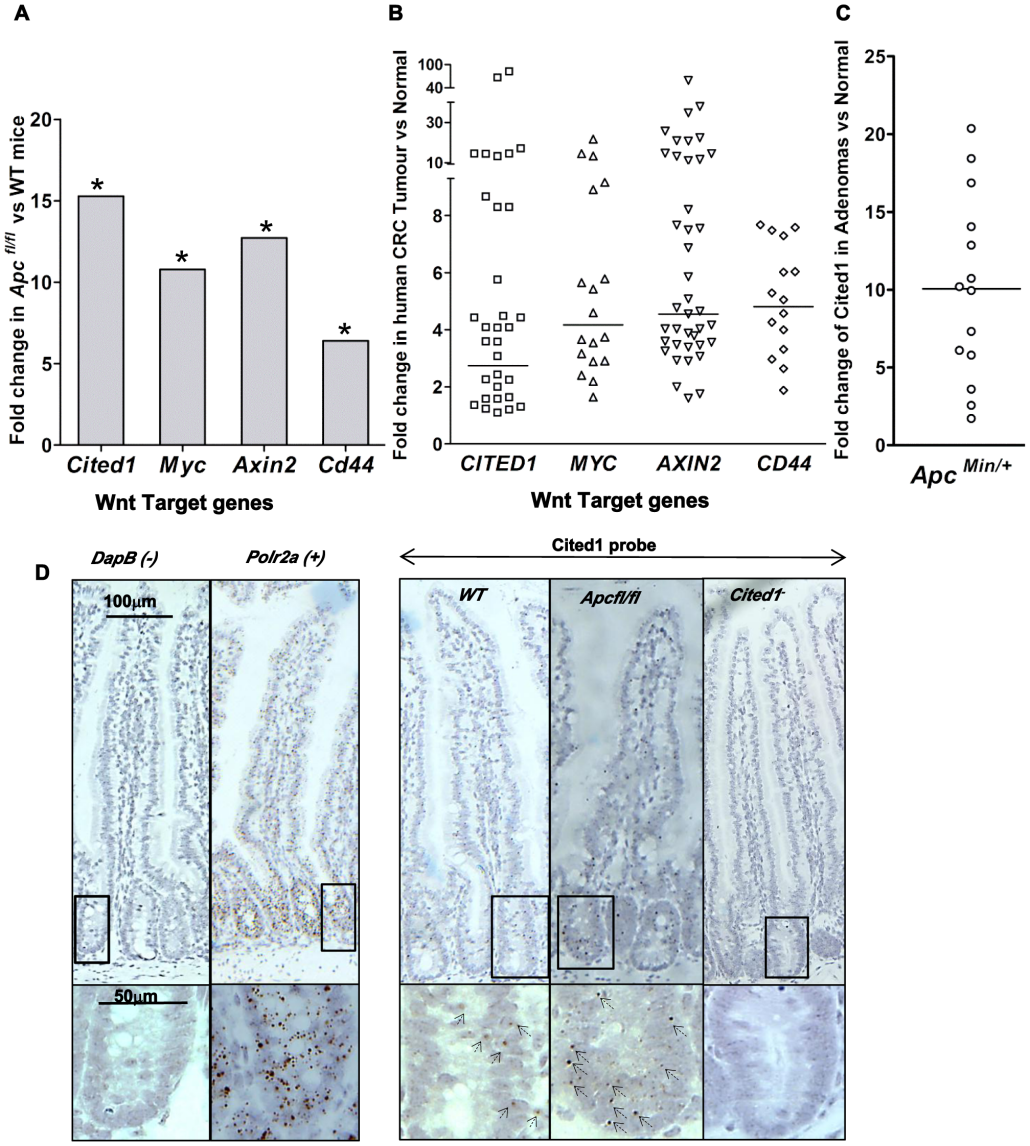
*Cited1* over-expression in *Apc^Min/+^* mice, *AhCre^+^Apc^fl/fl^* mice and human colorectal cancer. **A:** **** QPCR analysis of Wnt target genes 4 days after conditional deletion of *Apc* in the small intestinal. * p<0.05, Mann-Whitney U test, n = 3. **B:** Taqman qPCR analysis of gene expression in human CRC tumour tissue presented as fold change relative to adjacent normal tissue. The horizontal line indicates median fold change. p<0.01, Wilcoxon Signed Rank Test. **C:** Semi-QPCR analysis of *Cited1* expression in *Apc^Min/+^* adenomas. Three *Apc^Min/+^* mice were used and 3–6 tumours were taken from each individual, n = 14. (p<0.01, Mann-Whitney U test). **D:** *Cited1* in situ hybridization showing the increase in Cited1 expression in the *AhCre^+^Apc^fl/fl^* compare to *AhCre^+^WT* mouse. The staining is represented by single dots which correspond to the *Cited1* transcript. The low level of staining (compared to positive control probe Polr2a) of Cited1 probe is distributed throughout the crypt-villus structure in the *AhCre^+^WT* mouse and in *AhCre^+^Apc^fl/fl^* mouse with an increase in staining after loss of Apc. Absence of staining is observed in the *Cited1^−^* intestinal tissue and in the negative control probe DapB. Inset panels show magnifications of intestine of corresponding zone.

We have previously shown that deletion of Apc in the mouse intestine leads to nuclear β-catenin translocation and up-regulation of Wnt target genes, including *Cited1*, as scored by microarray analysis [3]. To confirm this upregulation, we analysed mouse intestinal epithelium from *AhCre^+^Apc^fl/fl^* and *AhCre^+^WT* (*WT: wild type*) mice which had been induced by intraperitoneal injection of β-napthoflavone 4 days previously [3]. Quantitative PCR analysis revealed significant upregulation of the Wnt targets *c-Myc*, *Axin2* and *Cd44* in the absence of *Apc*. Similarly, *Cited1* showed a 15-fold increase in expression (p<0.05, Figure 1A).

To determine if *CITED1* was also deregulated in human cancers, we performed a Taqman quantitative PCR on human colorectal tumour tissues. In comparison to paired normal tissues from the same patient, we observed over-expression of the human orthologues of the Wnt target genes *c-MYC*, *AXIN2*, *CD44* and *CITED1* (p<0.01, Figure 1B). These data demonstrate the potential transferability of our data from the acute *Apc* deletion mouse model to human colorectal carcinogenesis.

We next assessed *Cited1* levels in adenomas developing in the *Apc^Min/+^* mouse model of human colorectal cancer, which allows evaluation of the effects of loss of Apc function over the course of polyp development. Again, the level of *Cited1* expression was significantly increased in intestinal polyps from *Apc^Min/+^* mice compared to normal tissue from the same mouse (p<0.01, Figure 1C; Figure S1A). Detection of high levels of *Cited1/CITED1* expression in both human and murine tumours suggests that Cited1/CITED1 may play a role in intestinal tumourigenesis.

We performed in situ hybridization using a Cited1 probe designed against the deleted sequence in the *Cited1^−^* mouse (Figure 1D). We observed low levels of staining (compared to expression of the housekeeping gene Polr2a) throughout the crypt-villus structure in the *AhCre^+^WT* mouse intestine (*WT*) with a trend to higher levels within the crypt. There was no apparent specificity for the stem cell region at the base of the crypt or for any differentiated cell type. Consistent with QPCR data, we observed an increase in the level of staining throughout the crypt-villus structure of the *AhCre^+^Apc^fl/fl^* mouse.

### Loss of *Cited1* in *Apc^Min/+^* mice increases survival and reduces the number of intestinal adenomas

**Figure 2:**
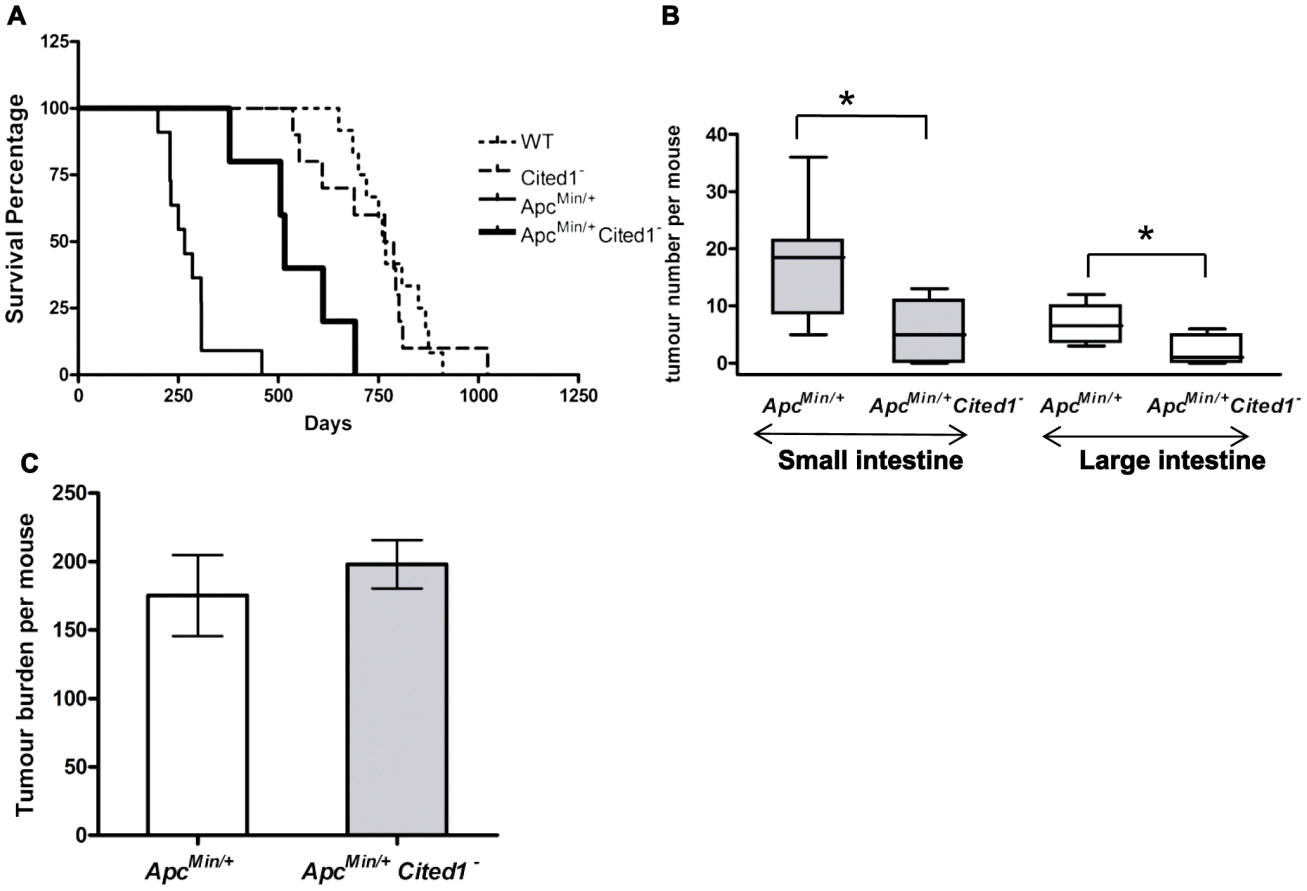
Loss of *Cited1* extends lifespan of *Apc^Min/+^* mice and reduces the number of intestinal adenomas. **A:** **** Kaplan-Meier plot of survival of *Apc^Min/+^* (thin continuous line), *Apc^Min/+^Cited1^−^* mice (thick continuous line), *Cited1^−^* (dashed line) and *WT* (dotted line). The median lifespan of *Apc^Min/+^* mice cohort was 230.5 days (n = 8), which was increased to 515 days in *Apc^Min/+^Cited1* ^−^ mice (n = 5) (p = 0.001 Log-Rank test). The median lifespan of *Cited1^−^* was 766 days (n = 10) and is similar to *WT* with a lifespan of 760 days (n = 12) (p = 0.749 Log-Rank test). **B:** In both small intestine (grey boxes) and large intestine (blank boxes), tumour numbers were reduced in *Apc^Min/+^Cited1^−^* mice (n = 5) compared to *Apc^Min/+^* mice (n = 8) (*p<0.05, Mann-Whitney U test). **C:** Tumour burden was calculated and represent the total tumour volume per mouse. There is no significant difference between *Apc^Min/+^* (white) and *Apc^Min/+^Cited1^−^* (Grey) (p>0.05, Mann-Whitney U test).

As *Cited1* is upregulated in colonic tumors we next asked whether deficiency of *Cited1* could inhibit intestinal adenoma formation in the *Apc^Min/+^* mouse. To achieve this, we crossed *Cited1* null (*Cited1^−^*) mice onto *Apc^Min/+^*. Given that *Cited1* is on the X-chromosome, we aged male cohorts of *Apc^Min/+^Cited1^−^* and *Apc^Min/+^* mice until they displayed symptoms of intestinal neoplasia (rectal bleeding and paling feet). The median lifespan of *Apc^Min/+^* mice was 230.5 days, which is increased to 515 days in the *Apc^Min/+^Cited1^−^* mice (Log-Rank p = 0.001, Figure 2A). *Cited1^−^* mice had a survival rate that was not significantly different to that of WT mice (Figure 2A).

We next counted the number of adenomas in the small and large intestine (Figure 2B). *Apc^Min/+^Cited1^−^* mice developed significantly less tumours compared to *Apc^Min/+^* in both the small intestine (5 versus 18.5 adenomas p<0.05) and the large intestine (1 versus 6.5 adenomas, p<0.05). The tumour distribution in the small intestine and the colon was analysed at ill health (Figure S1C). There was no significant difference in the percentage of tumours found in the duodenum or jejunum of the small intestine, or in the large intestine. However, we did observe a significant increase in the percentage of tumours found in the last part of the small intestine which corresponds to the human ileum (Figure S1C). Total tumour burden of *Apc^Min/+^Cited1^−^* mice was not significantly different from that of *Apc^Min/+^* (Figure 2C), and shared the same tubular morphology and degree of invasiveness, as assessed histologically by the frequency of invasion into the submucosa (52.8% High grade +47.16% Low grade in *Apc^Min/+^* vs 42.8% High grade +57.4% Low grade in *Apc^Min/+^Cited1^−^*, Chi-square x^2^ = 2.1, DF = 1, p>0.05). These data suggest that mice became symptomatic of disease when they had developed an equivalent tumour burden, but that in the *Cited1* mutant background this was significantly later and reflected fewer, but larger lesions at these later time points, hence implicating Cited1 in intestinal tumour initiation.

### *Cited1* deficiency modifies the phenotype observed immediately after Apc loss by increasing the number of Brdu positive cells and the size of the hyperplastic area

To address the mechanism underlying the reduction of adenoma formation in *Apc^Min/+^Cited1^−^* mice, we crossed *Cited1^−^* mice with mice conditionally mutant for *Apc*. We have previously demonstrated that we can achieve almost 100% recombination of the *Apc^fl/fl^* allele in the intestine using the β-napthoflavone inducible *AhCre* transgene to drive recombination [3]. Thus, *AhCre^+^WT*, *AhCre^+^Apc^fl/fl^*, *AhCre^+^Cited1^−^*, and *AhCre^+^Apc^fl/fl^Cited1^−^* mice were induced with β-napthoflavone and culled 4 days after the first injection to determine the role of *Cited1* immediately following deletion of Apc. To confirm the level of *Apc^fl/fl^* recombination we used quantitative RT-PCR and again found that 100% of the PCR products obtained were from the recombined *Apc* allele (Figure S1B). We also confirmed *Cited1* deficiency in *Cited1^−^* mice using RT-PCR. We observed a significant 3.81 fold difference decrease in *Cited1* expression in *AhCre^+^Cited1^−^* compare to *AhCre^+^WT*. The small difference observed is most likely due to the low level of expression of Cited1 in the intestine [21]. Due to the increased level of *Cited1* expression after loss of Apc, *Cited1* deficiency is more noticeable in the intestinal epithelial cells of *AhCre^+^Apc^fl/fl^Cited1^−^* mice which showed a 277.81 fold decrease compared to *AhCre^+^Apc^fl/fl^* mice (p<0.05 Mann-Whitney U test).

**Figure 3:**
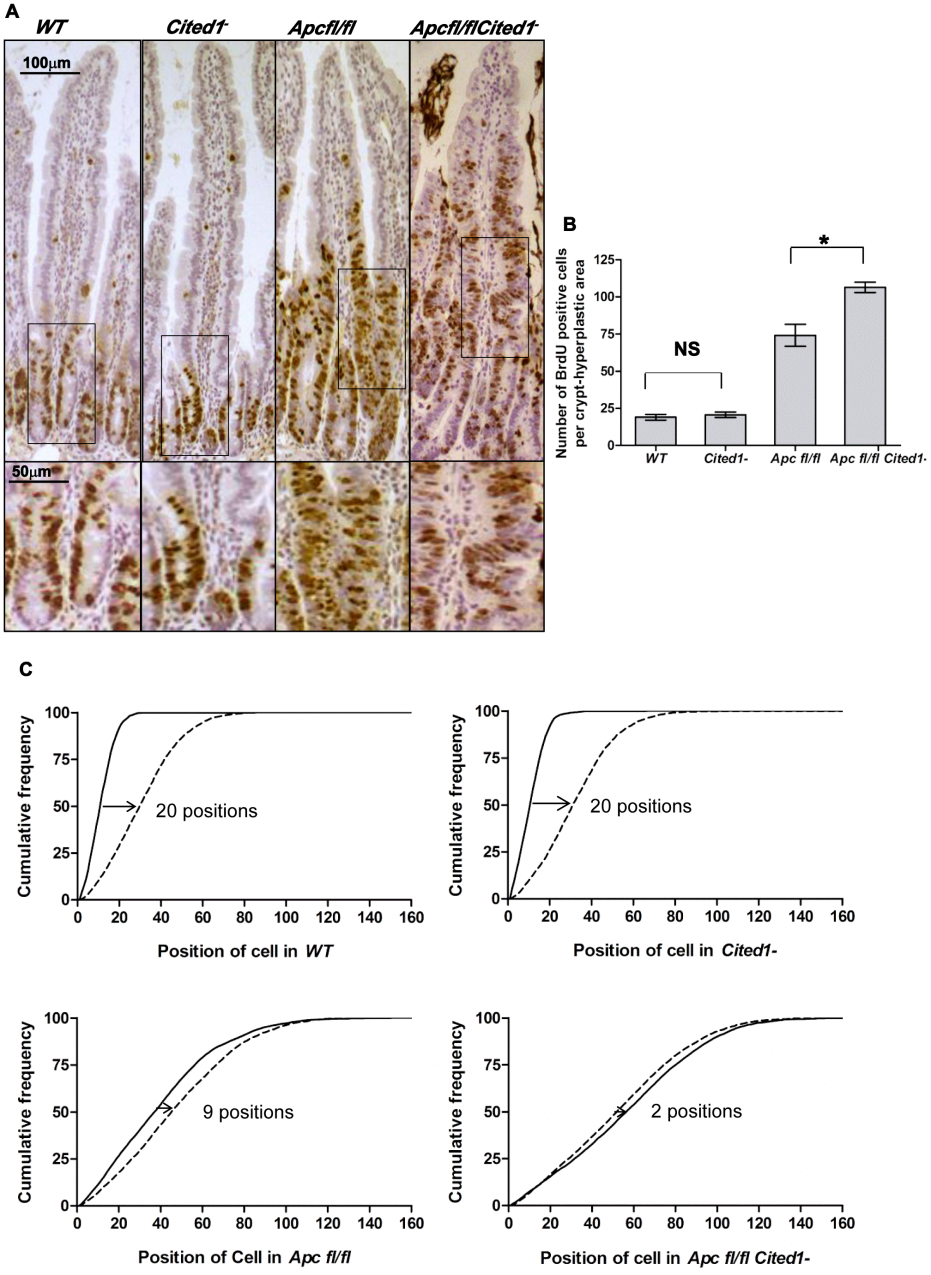
*Cited1* deficiency increases enterocyte proliferation and decreased cell migration in *AhCre^+^Apc^fl/fl^* mice. **A:** **** Cell proliferation assessed by IHC (immunohistochemistry) in *AhCre^+^WT* (*WT*), *AhCre^+^Cited1^−^*, (*Cited1^−^*) *AhCre^+^Apc^fl/fl^(Apc^fl/fl^)* and *AhCre^+^Apc^fl/fl^Cited1^−^(Apc^fl/fl^Cited1^−^)* mice. Bottom panels show magnifications of intestine of corresponding zone. **B:** Histograms showing the number of BrdU positive cells/crypt or hyperplastic areas. No significant difference in the localisation or number of BrdU positive cells between *AhCre^+^WT* and *AhCre^+^Cited1* ^−^ (p = 0.6625). Significant increase in the number of BrdU positive cell in *AhCre^+^Apc^fl/fl^Cited1^−^* compared to *AhCre^+^Apc^fl/fl^* (*p = 0.0404). **C:** Graphs showing the position of BrdU positive cells after 2 hrs (solid line) and 24 hrs (dashed line). The cumulative frequency represents the percentage of BrdU positive cells at a particular position from the bottom of the crypt or hyperplastic areas to the tip of the villus. The difference between the 2 hrs and 24 hrs distributions for a genotype was analysed with the Kolmogorov–Smirnov test. The distribution of Brdu positive cells from 2 hrs to 24 hrs varies significantly for all genotypes (p = 0.01) indicating cell migration. The migration of cells from 2 hrs to 24 hrs after Brdu labelling is similar in *AhCre^+^WT* compared to *AhCre^+^Cited1^−^* as shown by the distance between the 2 distributions (20 cell positions at the 50% cumulative frequency). In *AhCre^+^Apc^fl/fl^*, the distance is reduced to 9 cell positions indicating reduced migration compared to *AhCre^+^WT* (20 positions) and *AhCre^+^Cited1^−^* (20 positions). The migration is further reduced in *AhCre^+^Apc^fl/fl^Cited1^−^* as the 50% cumulative frequency position moved only 2 positions, compared to *AhCre^+^Apc^fl/fl^* (9 positions). *p<0.05; Statistical tests were done using Mann-Whitney U test (B) or Kolmogorov–Smirnov test (C); NS Non significant. (N = 3/genotype).

We have previously shown that the loss of *Apc* leads to an increase in proliferation and apoptosis and also to a loss of migration [3]. To analyse the effects of *Cited1* deficiency after *Apc* loss, we first counted the number of cells in S phase within the crypt or hyperplastic areas (formed after *Apc* loss). On day 4 after β-napthoflavone induction, mice were injected with BrdU to label cells in S-phase and culled 2 hrs later (Figure 3A). In *AhCre^+^WT* and *AhCre^+^Cited1^−^* mice the number of proliferating cells was not significantly different (*AhCre^+^WT*: 18.97 vs *AhCre^+^Cited1^−^*: 20.65 BrdU positive cells/Crypt; p>0.05. Figure 3B). However, the number of cells in S-phase was significantly increased in the hyperplastic areas of *AhCre^+^Apc^fl/fl^Cited1^−^* mice compared to *AhCre^+^Apc^fl/fl^* mice (*AhCre^+^Apc^fl/fl^*: 74.13 vs *AhCre^+^Apc^fl/fl^Cited1^−^*: 106.4 BrdU positive cells/area, p<0.05, Figure 3B) suggesting a role for *Cited1* in controlling cell proliferation in the context of active Wnt signaling.

**Figure 4:**
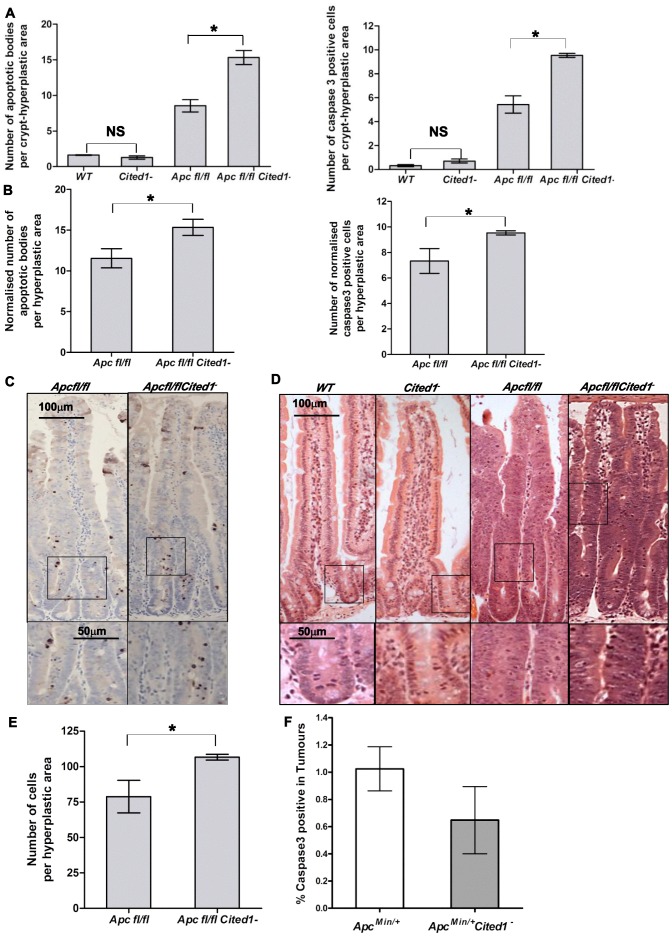
*Cited1* deficiency enhances increased apoptosis in *AhCre^+^Apc^fl/fl^* mice. **A:** **** Histograms showing the levels of apoptosis in each genotype. Apoptotic cells were scored by H&E staining (left) or cleaved-Caspase3 antibody (right). Both graphs show an increase in the number of apoptotic cells in *AhCre^+^Apc^fl/fl^Cited1^−^* compared to *AhCre^+^Apc^fl/fl^* (*AhCre^+^Apc^fl/fl^*<*AhCre^+^Apc^fl/fl^Cited1^−^*, Left: p = 0.0404; Right: p = 0.0259). No significant difference is observed between *AhCre^+^WT* and *AhCre^+^Cited1^−^* (*AhCre^+^WT* = *AhCre^+^Cited1^−^*, Left: p = 0.1914; right: p = 0.0952). **B:** The number of apoptotic cells were normalised to the number of total cells per hyperplastic area in *AhCre^+^Apc^fl/fl^* versus *AhCre^+^Apc^fl/fl^Cited1^−^*. After normalisation, the number of apoptotic cells is greater in *AhCre^+^Apc^fl/fl^Cited1^−^* compared to *AhCre^+^Apc^fl/fl^* (H&E: *AhCre^+^Apc^fl/fl^*<*AhCre^+^Apc^fl/fl^Cited1^−^*, p = 0.0404; cleaved-Caspase3: *AhCre^+^Apc^fl/fl^*<*AhCre^+^Apc^fl/fl^Cited1^−^*, p = 0.0259). **C:** cleaved-Caspase3 representative pictures showing an increase in apoptotic bodies in *AhCre^+^Apc^fl/fl^Cited1^−^* compared to *AhCre^+^Apc^fl/fl^*. **D:** Histology by H&E stained mouse intestinal sections in each genotype. Bottom panels show magnifications of intestine of corresponding zone. **E:** Scoring of epithelial cells per hyperplastic areas using the extent of BrdU labelling. Histograms show a significant increase in epithelial cell number in the hyperplastic areas from *AhCre^+^Apc^fl/fl^Cited1^−^* compared to *AhCre^+^Apc^fl/fl^* mice (p<0.05). *p<0.05; All statistical tests were done using Mann-Whitney U test ; NS Non significant. (n = 3/genotype). **F:** Histograms showing the levels of apoptosis (cleaved Caspase 3 antibody) in tumours of *Apc^Min/+^* (white) and *Apc^Min/+^Cited1^−^* mice (Grey). There is no significant difference between the 2 genotypes (p>0.05, Mann-Whitney U test).

We next analysed the histology on HE sections of the intestinal tissue from all the genotypes after β-napthoflavone induction. There were no gross changes in the crypt/villus architecture in *AhCre^+^WT* compared to *AhCre^+^Cited1^−^* mice, and induced *AhCre^+^Apc^fl/fl^Cited1^−^* mice had similar large aberrant crypts to those observed in *AhCre^+^Apc^fl/fl^* mice (Figure 4D).

Given our findings of decreased adenoma formation in *Apc^Min/+^* mice, we also examined the extent of the hyperplastic area within the crypts of *AhCre^+^Apc^fl/fl^* mice compared to *AhCre^+^Apc^fl/fl^Cited1^−^* mice as determined by the extent of BrdU labelling. Surprisingly, the number of cells in the hyperplastic area was greater in *AhCre^+^Apc^fl/fl^Cited1^−^* mice compared to *AhCre^+^Apc^fl/fl^* mice (*AhCre^+^Apc^fl/fl^*: 78 cells/area *vs AhCre^+^Apc^fl/fl^Cited1^−^* :106 cells/area, p<0.05, Figure 4E).

We next determined migration rates by comparing the position of cells 2 hrs and 24 hrs after BrdU labelling. The difference between the 2 hrs and 24 hrs distributions for a genotype was analysed with the Kolmogorov–Smirnov test. The distribution of Brdu positive cells from 2 hrs to 24 hrs varies significantly for all genotypes (p = 0.01) indicating cell migration. Enterocytes in *AhCre^+^WT* mice and *AhCre* ^+^ *Cited1^−^* mice migrate at the same rate (20 cell positions at the 50% cumulative frequency) whereas cells in both *AhCre^+^Apc^fl/fl^* mice and *AhCre^+^Apc^fl/fl^Cited1^−^* mice show greatly reduced migration rates (Figure 3C). Critically, although deletion of *Apc* in *AhCre^+^Apc^fl/fl^* mice results in strong suppression of migration (9 cell position migration), some movement of cells was detected in these samples (Figure 3C). By comparison, the absence of *Cited1* in *AhCre^+^Apc^fl/fl^Cited1^−^* mice, resulted in even less migration (2 cell position migration) than observed for enterocytes in *AhCre^+^Apc^fl/fl^* mice (Figure 3C). Together, these data demonstrate that *Cited1* deficiency further exacerbates both the proliferation and migration phenotypes of Apc loss in the intestine, which is surprising given that *Apc^Min/+^Cited1^−^* mice developed significantly less intestinal tumours than *Apc^Min/+^*mice. Consistent with these observations that proliferation is increased in *AhCre^+^Apc^fl/fl^Cited1^−^* mice, we also observed a decrease in the number of differentiated entoendocrine cells and goblet cells in these mice (Figure S5A–D).

We and others have previously demonstrated that the location and the number of paneth cells in the intestinal crypt are regulated by Wnt signalling [6], [22]–[24]. It is observed by the increased number of paneth cells after loss of Apc (Figure S5F) and the loss of positioning at the bottom of the crypt (Figure S5E–G). Consistent with our observations that the phenotype of *AhCre^+^Apc^fl/fl^* mice is enhanced upon deficiency of *Cited1* we also observe a change in position of the paneth cells in the hyperplastic areas of the *AhCre^+^Apc^fl/fl^Cited1^−^* compared to *AhCre^+^Apc^fl/fl^* (Figure S5G). This is most likely due to the increase in crypt size seen in the double mutant, which gives cells a bigger area to be distributed.

### *Cited1* deficiency enhances the apoptotic phenotype observed immediately after Apc loss

The increase in proliferation observed in the intestine following deletion of Apc is also associated with a dramatic increase in apoptosis [3]. We therefore examined if Cited1 was regulating apoptosis by counting apoptotic bodies in H&E sections and also scoring Caspase 3 staining. We observed no significant difference in apoptosis between *AhCre^+^WT* and *AhCre^+^Cited1^−^* mice, however, there was a significant increase in the number of apoptotic cells in *AhCre^+^Apc^fl/fl^Cited1^−^* mice compared to *AhCre^+^Apc^fl/fl^* mice, which was verified by both methods (Figure 4A).

As mentioned above, we observed an increase in the number of cells per hyperplastic area in the *AhCre^+^Apc^fl/fl^Cited1^−^* samples. To verify that the increase in cell death was not an artefact of the difference in the number of cells per area, we corrected for this difference between *AhCre^+^Apc^fl/fl^* and *AhCre^+^Apc^fl/fl^Cited1^−^* mice. The normalised data confirmed increased cell death in *AhCre^+^Apc^fl/fl^Cited1^−^* compared to *AhCre^+^Apc^fl/fl^* (p<0.05) after H&E counting (*AhCre^+^Apc^fl/fl^* : 11.53 apoptotic cells/area *vs AhCre^+^Apc^fl/fl^Cited1^−^*: 15.33 apoptotic cells/area, p<0.05) and after anti cleaved-Caspase3 staining (*AhCre^+^Apc^fl/fl^*: 7.32 apoptotic cells/area *vs AhCre^+^Apc^fl/fl^Cited1^−^*: 9.53 apoptotic cells/area, p<0.05) (Figure 4B–C). These data indicate that the increase in cell death is not proportional to the increase in cell proliferation. Therefore, *Cited1* deficiency in a Wnt perturbed background accentuates the apoptotic response.

Scoring of Caspase3 positive cells revealed no change in the number of apoptotic cells in the intestinal tumors of *Apc^Min/+^Cited1^−^* mice compared to *Apc^Min/+^* mice at time of death (Figure 4F). Therefore, the increased apoptosis we observe in the absence of Cited1 is only manifested in the context of acute Wnt activation, which underlines the role of Cited1 in restraining tumour initiation, and also implies that in those tumours that do develop in the absence of *Cited1*, they have developed alternate mechanisms to restrain the Wnt pathway.

### *Cited 1* regulates several pathways including the Wnt signaling pathway

We next wished to investigate the mechanism through which Cited1 may be modifying Wnt driven tumorigenesis. One possibility is a direct effect upon Wnt signaling, and in support of this, Cited1 has previously been shown to be able to bind to β-catenin and consequently inhibit Wnt induced transcription during *Xenopus* development [5]. Two potential TCF-4 sites were identified in the *Cited1* promoter region (ctttgt and cattgaa in the 2 kb prior exon1). This implicates *Cited1* in the control of the Wnt pathway, however this is not the only pathway known to be altered by *Cited1*. Cited1 has been shown to bind to the p300/CBP coactivators and also to Smad4, thereby enhancing their transcriptional activity [11], [25]. To analyse the effects of *Cited1* deficiency on various transcriptional pathways we performed a microarray analysis using the Affimetrix Chip 430 2.0 and AffylmGUI software [26]. We then submitted our microarray data to ingenuity pathway analysis software (IPA) to identify pathways significantly affected by *Cited1* deficiency.

In the *AhCre^+^WT* after additional loss of *Cited1*, a number of signaling pathways identified by IPA analysis were found to be affected, amongst them: P53 (p = 6.87×10^−6^, ratio = 0.146), PI3K/AKT (p = 6.74×10^−6^, ratio = 0.114), Pten (p = 7.7×10^−4^, ratio = 0.097); Wnt (p = 1.08×10^−1^, ratio = 0.057); and TGFβ (p>0.1, ratio = 0.034). Several targets were analysed by QPCR including *c-Myc*, *Axin2*, *CD44*, *Sox4*, *p53*, *Pten*, *Akt1*, and *Smad4* but none were found to be significantly deregulated (N = 6, p>0.05; Mann-Whitney).

**Figure 5:**
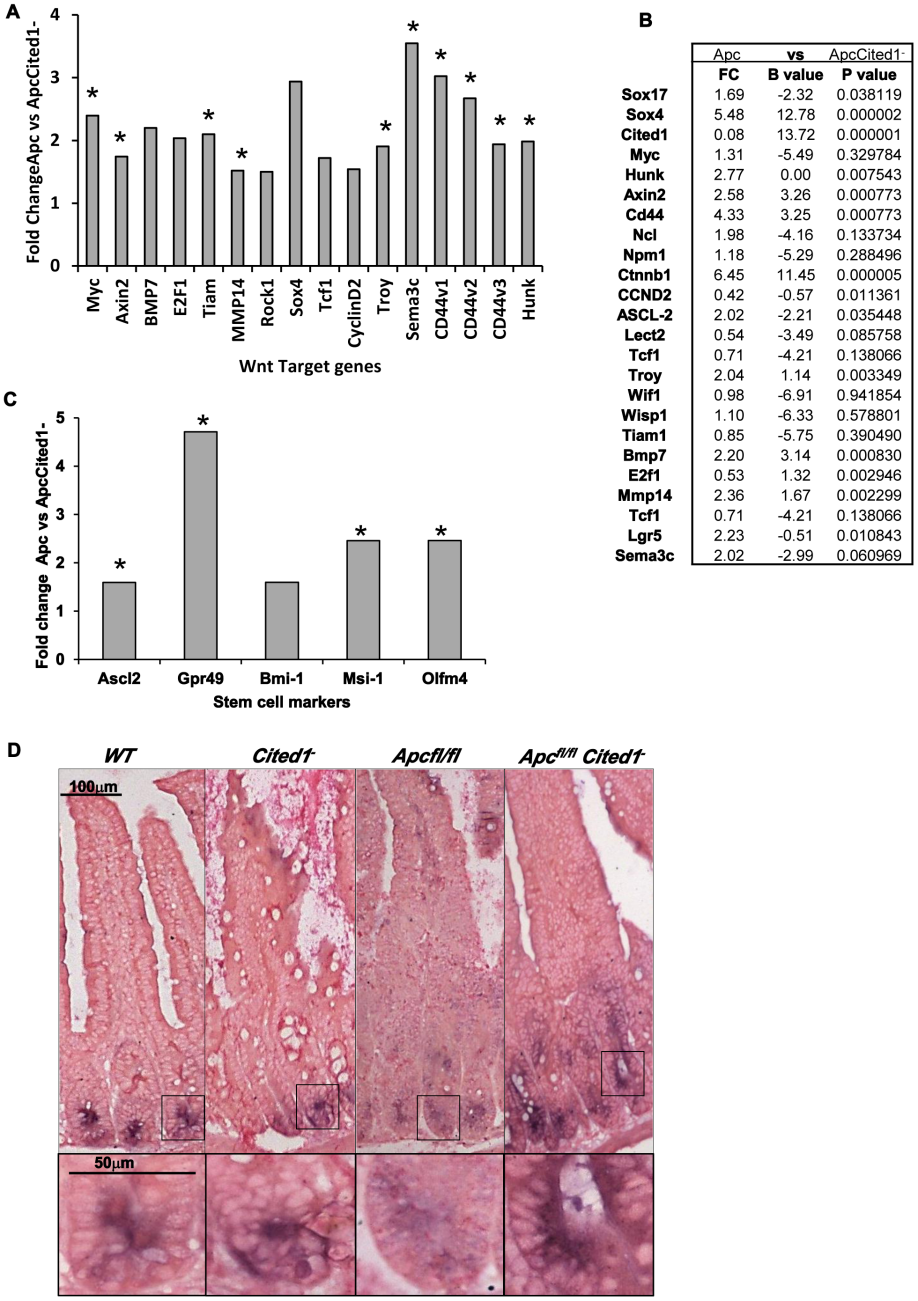
Expression levels of Wnt target genes after loss of *Cited*. **A:** **** Fold change of Wnt target gene expression in the small intestinal epithelium of *AhCre^+^Apc^fl/fl^Cited1^−^* compared to *AhCre^+^Apc^fl/fl^* mice measured by QRT-PCR, *p<0.05 Mann Whitney U test. **B:** Microarray analysis showing up-regulation of Wnt target genes with Wnt Key players in *AhCre^+^Apc^fl/fl^Cited1^−^* compared to *AhCre^+^Apc^fl/fl^*: Fold changes (FC) are presented with correspondent P value and B value (B statistic is lod score). **C:** Fold change of stem cell markers expression in the small intestinal epithelium of *AhCre^+^Apc^fl/fl^Cited1^−^* compared to *AhCre^+^Apc^fl/fl^* mice measured by QRT-PCR, *p<0.05 Mann Whitney U test. **D:** In situ Hybridization (ISH) analysis of *Olfm4* in intestinal epithelial cells of all 4 genotypes. Inset panels show magnifications of intestine of corresponding zone.

Several signaling pathways identified by IPA analysis were affected in *AhCre^+^Apc^fl/fl^Cited1^−^* mice compared to *AhCre^+^Apc^fl/fl^* mice, including: P53 (p = 3.5×10^−8^, ratio = 0.177); PI3K/AKT (p = 2.62×10^−5^, ratio = 0.107); Pten (p = 7.22×10^−4^, ratio = 0.097), Wnt (p = 5.4×10^−2^, ratio = 0.063); and TGFβ (p = 2.22×10^−1^, ratio 0.056). The validity of the IPA analysis was subsequently verified by QPCR. We analysed several targets from these pathways by QPCR, and found significant upregulation of *p53*, *Runx1*, *Sox4* (Figure S2C) and a number of Wnt targets known to be deregulated in the intestines of *AhCre^+^Apc^fl/fl^* mice [3] or listed as Wnt target genes in the Nusse webpage (http://www.stanford.edu/group/nusselab/cgi-bin/wnt/target_genes) (Figure 5A–B). 10 Wnt target genes, including *c-Myc*, *Axin2*, and *CD44* were confirmed by QPCR to be significantly up-regulated in *AhCre^+^Apc^fl/fl^Cited1^−^* mice compared to *AhCre^+^Apc^fl/fl^* mice (Figure 5A). Three additional transcripts were analysed by microarray analysis that have previously been identified as key players in the Wnt pathway (Nucleophosmin, Nucleolin, and β-catenin respectively: [27]–[29]). These were also found to be upregulated in *AhCre^+^Apc^fl/fl^Cited1^−^* mice compared to *AhCre^+^Apc^fl/fl^* mice. These data indicate that *Cited1* inhibits several signaling pathways, including the Wnt pathway following *Apc* loss.

### Stem cell markers are upregulated after loss of *Cited1*

The Wnt signalling pathway has been shown to play a critical role in intestinal homeostasis which includes stem cells maintenance. Because *Cited1* loss leads to a deregulation of the Wnt pathway and due to the potential role of Cited1 in the stem cell niche in the cap mesemchyme in the developing kidney [18], we analysed the effect of *Cited1* deficiency in the intestine. RT-QPCR analysis revealed a significant upregulation of several stem cell markers (*Gpr49*, *Ascl2*, *Musashi* and *Olfm4*) in *AhCre^+^Apc^fl/fl^Cited1^−^* tissues compared to *AhCre^+^Apc^fl/fl^* controls (Figure 5C). We also performed ISH for the surrogate marker of *Lgr5*, *Olfm4* (Figure 5D). In *AhCre^+^Wt* and *AhCre^+^Cited1* ^−^ mice the location of *Olfm4* expressing cells is confined to the stem cell niche at the base of the crypts. In *AhCre^+^Apc^fl/fl^* mice, *Olfm4* expressing cells were distributed throughout the hyperplastic area. In *AhCre^+^Apc^fl/fl^Cited1^−^* mice *Olfm4* expressing cells are also mislocalised throughout the aberrant crypts but expression is increased, consistent with our RT-QPCR data of the same tissue (Figure 5C).

### *Cited1* influences the level of the active form of β-catenin in the small intestine

**Figure 6:**
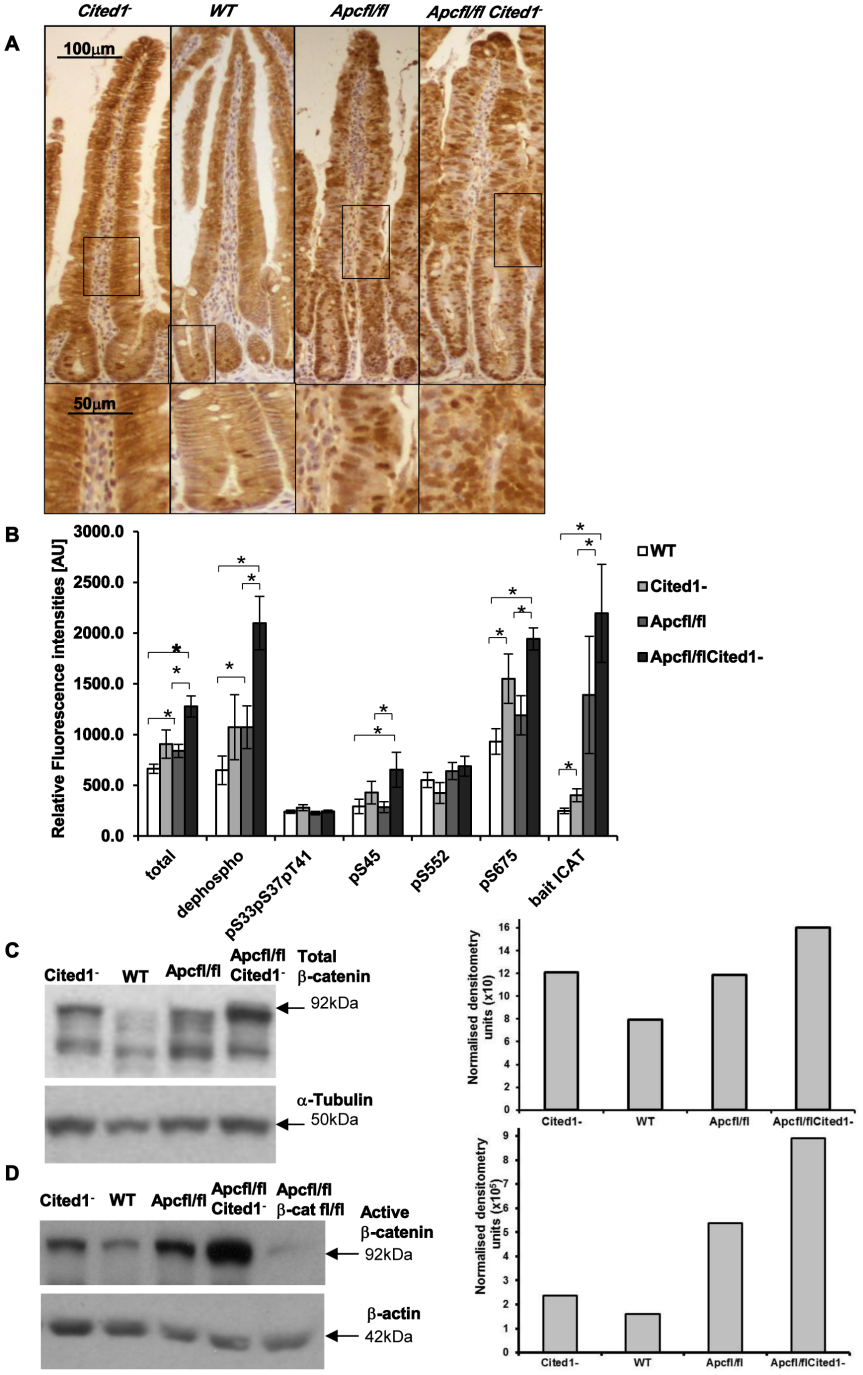
Level of the active form of β-catenin in the small intestine. **A:** Immunohistochemistry for total β-catenin in each genotype. Inset panels show magnifications of intestine of corresponding zone. **B:** Small intestine epithelial cell extracts from *AhCre^+^WT* (WT; n = 14), *AhCre^+^Cited1^−^* (Cited1^−^; n = 7); *AhCre^+^Apc^fl/fl^* (Apc^fl/fl^; n = 12); *AhCre^+^Apc^fl/fl^Cited1^−^* (Apc^fl/fl^Cited1^−^) were analysed for the status of β-catenin using a suspension bead array assay panel. Total β-catenin, dephospho β-catenin (S33, S37 and T41), phosphorylation at S33, S37, and T41, phosphorylation at S45, phosphorylation at S552 and phosphorylation at S675 were analysed by using respective capture antibodies in multiplexed sandwich immunoassays. Free β-catenin (non-complexed) was measured by µGST pull-down assays using GST-ICAT as bait protein. Signal intensities are displayed in relative fluorescence units [AU] (mean+SE). *p<0.05; All statistical tests were done using Mann-Whitney U test. **C:** Western blot analysis of the Total form of β-catenin in each genotype. The histogram represents the densitometry analysis of the total β-catenin immuno-blot normalised to the internal control α-tubulin, showing an increase in the level of total β-catenin in *AhCre^+^Apc^fl/fl^Cited1^−^ (Apc^fl/fl^Cited1^−^)* compared to *AhCre^+^Apc^fl/fl^(Apc^fl/fl^)* and *AhCre^+^Cited1^−^* also show an increase compare to *AhCre^+^WT*. **D:** Western blot analysis of the active form of β-catenin using dephospho-β-catenin (dephosphorylated on Ser37/Thr41, Clone 8E7, Millipore) antibody in each genotype. The histogram represents the densitometry analysis of the dephospho-β-catenin immuno-blot normalised to the internal control β-actin, showing an increase in the level of dephos-β-catenin in *AhCre^+^Apc^fl/fl^Cited1^−^ (Apc^fl/fl^Cited1^−^)* compared to *AhCre^+^Apc^fl/fl^(Apc^fl/fl^)*. The sample of each genotype is pooled from 3 to 7 mice in the cohort. *AhCre^+^Apc^fl/fl^β-cat^fl/fl^* (*Apc^fl/fl^β-cat^fl/fl^*) is used as a negative control for dephospho-β-catenin.

Loss of Apc has been shown to drive an increase in total β-catenin and more importantly a re-localisation of the active form of β-catenin to the nucleus [3]. To test if *Cited1* deficiency modified this phenotype, we analysed the localisation of total β-catenin in the small intestine by immuno-histochemistry (Figure 6A). We observed a normal pattern of localisation in both *AhCre^+^WT* and *AhCre^+^Cited1^−^* mice consistent with previous findings [30]. Upon deletion of Apc, we observed nuclear translocation of β-catenin in the aberrant crypts of both *AhCre^+^Apc^fl/fl^* and *AhCre^+^Apc^fl/fl^Cited1^−^* mice (Figure 6A) indicative of de-regulated Wnt signalling.

β-catenin regulates important cellular functions such as transcription and adhesion [31], and the cellular concentration and phosphorylation status of β-catenin has been shown to impact on these functions [31], [29]. As we observe an increase in the transcription of several Wnt target genes in *AhCre^+^Apc^fl/fl^Cited1^−^* mice we examined the level of total β-catenin, the extent of phosphorylation at multiple sites and the ratio of transcriptionally active free β-catenin in purified intestinal epithelial cells (Figure 6B) as previously described [32]. First, we observed a significant increase in total β-catenin accompanied by an increase in the active form of β-catenin (dephosphorylation at pS33, pS37, pT41 sites) in *AhCre^+^Apc^fl/fl^* and *AhCre^+^Apc^fl/fl^Cited1^−^* mice compared to *AhCre^+^WT* and very importantly in *AhCre^+^Apc^fl/fl^* mice compared to *AhCre^+^Apc^fl/fl^Cited1^−^* mice.

These data were confirmed by western blot analysis using an antibody raised against total β-catenin (Figure 6C) or against the active form of β-catenin (dephosphorylated sites pS33, pS37, pT41) (Figure 6D) and were verified with a second antibody against dephosphorylated β-catenin (Figure S2A–B). There was no significant difference in the phosphorylated (inactive) form of β-catenin (phosphorylated β-catenin at pS33, pS37, pT41 sites is degraded as a mechanism of regulating Wnt signalling) between all genotypes (Figure 6B). β-catenin phosphorylated at pS45 (phosphorylated by casein kinase Iα as part of degradation pathway) is significantly increased in *AhCre^+^Apc^fl/fl^Cited1^−^* compared to *AhCre^+^WT* and *AhCre^+^Apc^fl/fl^*.

Given that β-catenin can also be phosphorylated by Protein Kinase A (PKA) at Ser552 and Ser675 which acts to inhibit ubiquitination and therefore increase levels of active β-catenin [33], we also analysed levels of pS552 and pS675 and found phosphorylation at S675 significantly increased in *AhCre^+^Apc^fl/fl^Cited1^−^* tissues compared to *AhCre^+^WT* and *AhCre^+^Apc^fl/fl^*, demonstrating the ability of Cited1 to regulate β-catenin at multiple sites (Figure 6B).

We also measured the intracellular free β-catenin (active β-catenin) levels by pull-down with a GST-fusion protein of the inhibitor of β-catenin and TCF-4 (ICAT). We observed a significant increase in free β-catenin in *AhCre^+^Apc^fl/fl^* and *AhCre^+^Apc^fl/fl^Cited1^−^* mice compared to *AhCre^+^WT* and noticeably a significant increase in *AhCre^+^Apc^fl/fl^Cited1^−^* compared to *AhCre^+^Apc^fl/fl^* mice (Figure 6B). These data support our findings above which indicate that Cited1 deficiency increases the levels of active dephosphorylated β-catenin.

These data demonstrated that the active dephosphorylated form of β-catenin in purified intestinal epithelial cells is markedly increased upon Cited1 deficiency. Although the level of dephosphorylated β-catenin is increased in *Cited1^−^* intestinal cells compared to *WT* mice, it is below that observed in the *AhCre^+^Apc^fl/fl^* intestinal cells (Figure 6B, 6D). As Cited1^−^ mice do not develop any intestinal phenotypes such as hyperproliferation this suggests that the level of Wnt activation in Cited1^−^ mice is below the critical threshold required to induce neoplasia [34]. However, when Apc is deleted in Cited1 deficient mice (*AhCre^+^Apc^fl/fl^Cited1^−^*) the level of dephosphorylated β-catenin is greater than that observed with Apc loss alone, thus providing an explanation for the increased transcription of Wnt target genes observed in these mice (Figure 5A).

## Discussion

Colorectal cancer is driven by a multiplicity of different biochemical pathways, however, key amongst these is the Wnt pathway, which we and others have previously shown to activate a set of *c-Myc* dependent genes which are critical for the early stages of colorectal cancer [6], [35]. One of these genes is *Cited1*, which has been found to interact at the protein level with β-catenin and thereby negatively regulate β-catenin transcription [5]. Its relevance to carcinogenesis has already been described as *Cited1* up-regulation has been observed in various cancers [14], [36], [37]. Here, we have extended those observations and find that *CITED1* is significantly up-regulated in colorectal tumours from patients and in intestinal adenomas developing in the *Apc^Min/+^* mouse model [38]. We also previously found *Cited1* to be over-expressed in intestinal epithelial cells immediately following deletion of the Wnt regulator gene Apc in *AhCre^+^Apc^fl/fl^* mice in a *c-Myc* dependent manner [6]. These data establish *Cited1* as an immediate Wnt target gene in the intestine.

On the basis of these data we hypothesised that Cited1 might control β-catenin activity and thereby modulate Wnt signaling activation and its effects on colorectal tumorigenesis. To investigate this, we used microarray analysis and quantitative PCR studies to show that loss of *Cited1* on an *Apc* deficient background does indeed impact upon a range of oncogenic signaling pathways, including Wnt. Our array data therefore show multiple effects of *Cited1* deficiency including negative regulation of the Wnt-pathway.

To investigate the requirement of *Cited1* during Wnt induced tumourigenesis, we analysed the effects of deletion of *Cited1* in two well characterised mouse models of Wnt signaling activation; the *Apc^Min/+^* mouse model of colorectal tumourigenesis and the *AhCre^+^Apc^fl/fl^* mouse, a conditional model of *Apc* loss in which the immediate phenotypic consequences of Apc deletion can be studied [3]. Surprisingly, we obtained the apparently paradoxical result that although *Apc^Min^* ^/+^ *Cited1* ^−^ mice developed fewer intestinal tumours (associated with an increased life-span) than *Apc^Min^* ^/+^ mice, the phenotypes induced upon conditional loss of Apc (including perturbed cell proliferation, apoptosis, differentiation and migration) were enhanced, rather than diminished, with additional loss of *Cited1*. Of note, we observed reduced capacity to differentiate (reflected by a reduced number of goblet cells and enteroendocrine cells), but no difference in total paneth cell numbers, although we did observe a difference in the positioning of paneth cells, which may well reflect differences in the Wnt signalling environment.

Our studies, suggest that a possible explanation for this apparent paradox is that the hyper-activated Wnt phenotype that occurs in the absence of *Cited1* includes increased apoptosis. Several studies in cell culture systems already support such a model. For example, it has been reported that overexpression of β-catenin when transfected into cell lines leads to a 3–4 fold increase in cell death [39]. In addition, it has been demonstrated that high levels of c-Myc induce apoptosis *in vivo* [40]. This is consistent with our observations that c-Myc is overexpressed immediately following deletion of Apc in the intestine and that levels are significantly increased further with additional absence of *Cited1*. We interpret our data to indicate that the increase in apoptosis may counteract the increase in proliferation to the extent that the overall effect is reduced development of Wnt transformed cells and consequently inhibition of tumourigenesis.

The mechanism underlying such hyper-activation of Wnt signaling appears to be at least in part mediated through increased levels of dephosphorylated β-catenin, which we found to be up-regulated in *AhCre^+^Apc^fl/fl^Cited1^−^* tissue compared to comparable *AhCre^+^Apc^fl/fl^* tissue at both the transcriptional and protein levels. Thus, we found increased levels of the dephosphorylated forms of β-catenin (T41, S33, S37). These sites when phosphorylated are involved in the degradation of β-catenin by the proteasome pathway [31]. This is accompanied by an increase in the levels of phosphorylation at serine 675 which has been shown to be phosphorylated by protein kinase A (PKA) and which has been shown to lead to inhibiting of ubiquitination of β-catenin causing its accumulation and subsequent Wnt signalling activation. We therefore show that Cited deficiency increases the pool of active β-catenin, consistent with the enhanced Wnt pathway activation we observe. It does however remain possible that *Cited1* may in addition be mediating its effects downstream of β-catenin.

We cannot rule out the possibility that the other pathway changes we observe are responsible for the reduction in tumourigenesis in *Apc^Min/+^Cited1^−^* mice, as the effects of loss of *Cited1* are not exclusive to the Wnt pathway. We also cannot rule out that the effects we observe may be secondary to *Cited1* deletion. For example, it is possible that some of the changes we observe may be due directly to the upregulation of c-Myc rather than a direct consequence of *Cited1* loss. Functional delineation of the precise relevance of all the changes we observe requires multiple crosses onto the relevant pathways to probe such dependency. Finally, *AhCre^+^WT*, *AhCre^+^Apc^fl/fl^*, *AhCre^+^Cited1^−^*, and *AhCre^+^Apc^fl/fl^Cited1^−^* mice were maintained on an outbred background, and because the comparisons of genotypes within the same littermates was restricted due to the small number of litter size, we cannot completely rule out the effect of gene modifiers on the *Cited1* loss phenotype.

**Figure 7:**
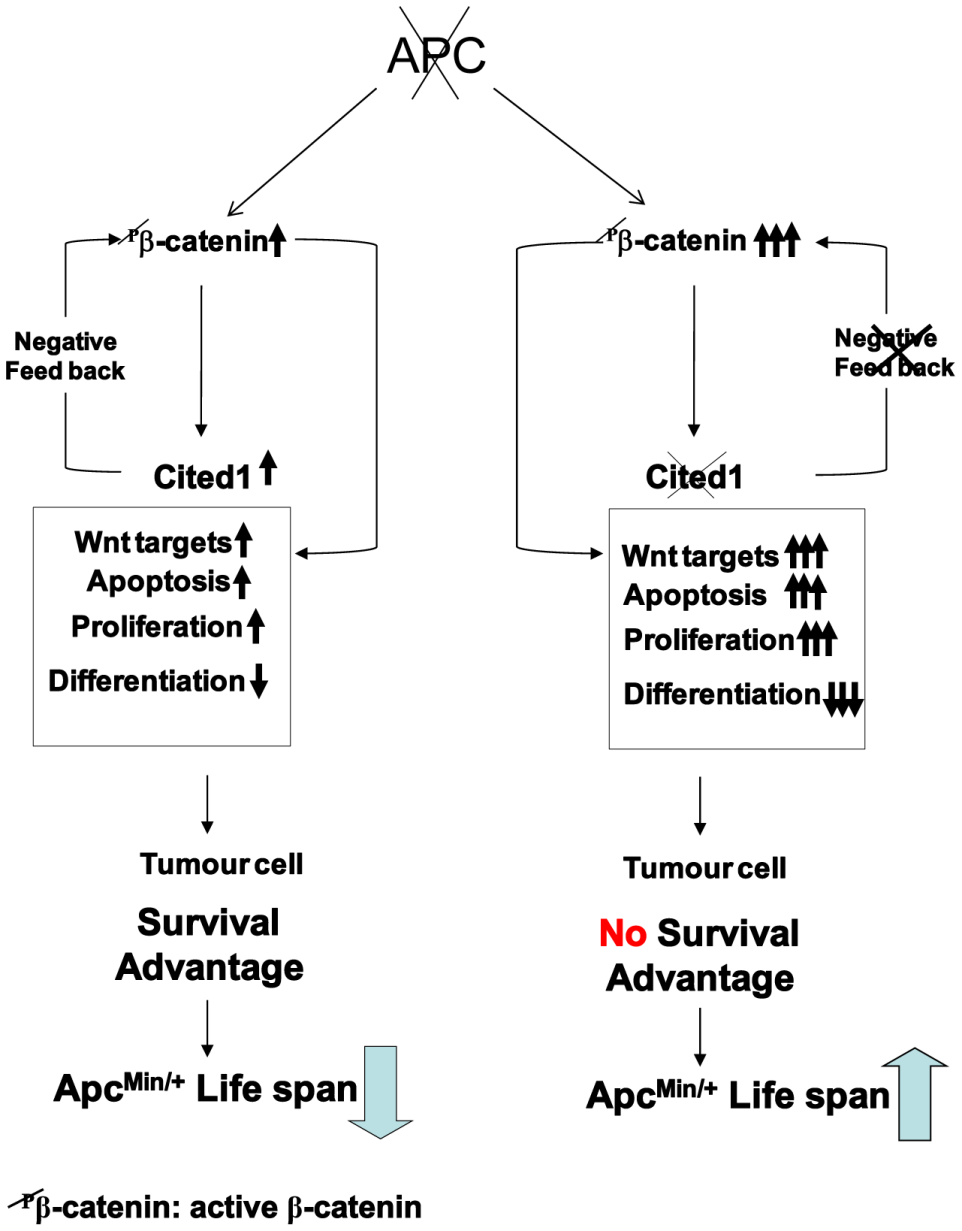
Schematic diagram of a “just right” model of *Cited1* action on colorectal tumourigenesis. Loss of function of Apc is accompanied by multiple changes in gene expression, including upregulation of active β-catenin (dephospho-β-catenin), activation of Wnt and *Cited1*. Hence, immediately following deletion of Apc, *Cited1* normally restrains the Wnt pathway at the level of β-catenin. We observe a range of rapid phenotypic changes. These include increase in proliferation and apoptosis and loss of differentiated cell types, also reduced migration, leading to the preferential retention of Apc deficient cells. These changes may all be considered pro-tumourigenic, leading to survival advantage of the tumour cell and decreased survival of the *Apc^Min/+^* mouse compared to *WT*. Additional *Cited1* deficiency leads to hyper-activation of Wnt signaling including upregulation of active β-catenin and an exaggerated Wnt phenotype including elevated proliferation, a further loss of cell differentiation, and most importantly increased cell death. The net effect of these changes is an increase in *Apc^Min/+^* survival. This restraint imposed by *Cited1* is consistent with a requirement for *Cited1* to constrain Wnt activity to a level commensurate with optimal adenoma formation and maintenance, and provides one mechanism for tumour repression in the absence of *Cited1*.

Our current primary hypothesis is that *Cited1* deficiency mediates its effects upon adenoma formation primarily through the apparently paradoxical derepression of the Wnt pathway. This result is however consistent with a “just right model” wherein a specific level of Wnt signaling activity is required for maximal tumour development and those levels of Wnt signaling above or below this level compromise tumour growth [41]. This model is further supported by recent studies on a novel mutant Apc mouse (Apc^1322T^), which has reduced Wnt signaling compared to *Apc^Min/+^* littermates, but surprisingly develops significantly more intestinal tumours [42]. Recently it was shown by Leedham et al [43] that in normal mouse intestine, stem cell markers and Wnt target genes are expressed in a physiological gradient compatible with normal intestinal homeostasis. Pathological activation of Wnt activity using the *Ctnnb1^Δex3^* mouse model led to variable gradients in stem cell number and Wnt signalling activity which influenced tumour susceptibility, with regional differences in tumour predisposition throughout the length of the intestinal tract. This data, which supports the just right model hypothesis, may explain the variation we observe in the tumour distribution in the ileum between the *Apc^Min/+^* and *Apc^Min/+^Cited1^−^* mice models. These observations clearly show that there is not a simple linear relationship between Wnt pathway activity and tumour burden. Our data is consistent with another version of this “just right” concept where perturbation of *Cited1* leads to increased dephosphorylated β-catenin and hyper-activation of the Wnt pathway to a level that is incompatible with maximum tumour growth (Figure 7). Notably, this relationship appears specific to the intestine as similar analysis of kidney tumorigenesis in these mice showed no effect of Cited deficiency (Figure S3). Furthermore, to define the precise relationship between Wnt levels and tumourigenicity will require mouse modelling experiments in which Wnt activity is precisely regulated at numerous levels.

Wnt/beta-catenin signalling plays a key role in the homeostasis of the intestinal epithelium and its role in the fate and maintenance of the stem cell compartment have been clearly demonstrated [44]. Our data clearly show that Cited1 is an immediate target of Wnt signalling and is an important regulator of the Wnt pathway. The loss of *Cited1* has a direct impact on stem cell status in the small intestine as we have found several stem cell markers to be upregulated including *Lgr5* (*Gpr49*), *Musashi* and *Olfm4*. These alterations in expression could be a direct consequence of the ‘hyper’ activation of the Wnt pathway we observe after combined loss of *Apc* and *Cited1*. This would implicate Cited1 as an important player in Wnt dependant stem cell maintenance in the small intestine. This has been already suggested in the developing kidney where Cited1 may contribute to the maintenance of the self-renewing capping mesenchyme [18]. By regulating the Wnt pathway, Cited1 may be an important regulator of the self-renewal compartment in the crypt of the small intestine.

Our data show that *Cited1* deficiency represses tumourigenesis. The consequences of *Cited1* deficiency are diverse, but in particular impact upon Wnt pathway activity. We propose a model whereby loss of *Cited1*, in the context of deregulated Wnt signaling, hyper-activates the Wnt pathway resulting in apoptosis of Wnt induced transformed cells and thus inhibits tumourigenesis. As *Cited1* mice are fertile and viable this suggests that *Cited1* represents a possible target for therapeutic intervention, where Cited1 inhibition induces cytotoxic effects due to very high Wnt signalling.

## Materials and Methods

### Human colorectal cancer tissue RNA samples

Total RNA samples from patient colorectal tumour tissues were obtained from the Cancer Tissue Bank Research Centre (CTBRC). All colorectal cancer tissues and adjacent uninvolved colonic mucosa were obtained from surgically removed specimens with informed patient consent. Uninvolved colonic mucosa was generally 5–10 cm away from the malignant tissue.

### Mouse colonies

All experiments were performed under the UK Home Office guidelines. Mice were obtained and genotyped as follows: *Cited1* null (*Cited1^−^*) [9]; *Apc^Min/+^* [38]; *AhCre* transgene (*AhCre+*) [45]; *Apc^580S^* allele [46]; *β-cat^fl/fl^* [47]; *Apc^Min/+^* and *Apc^Min+^Cited1^−^* mice were maintained on an inbred C57BL/6J background and were confirmed as congenic for the C57BL/6 *Mom-1* allele via PCR analysis. Mice were sacrificed at ill-health. Intestine were fixed in Methacarn (methanol-chloroform-glacial acetic acid [4∶2∶1]), and the lesion numbers were scored macroscopically.

To study the role of Cited1 after the early loss of *Apc*, *AhCre^+^WT*, *AhCre^+^Apc^fl/fl^*, *AhCre^+^Cited1^−^* and *AhCre^+^Apc^fl/fl^Cited1^−^* mice were generated and maintained on an outbred background. Cre activity was induced by three intraperitoneal injections of 80 mg/kg β-naphthoflavone within 24 h and mice were taken Day4 or Day5 later. Tissues analysed were from age (8–12 weeks), sex (males), background and genotype matched animals, however these were not always littermates.

### Assaying apoptosis, number of cells per crypt, S-phase labelling *in vivo* and migration

Apoptosis was scored from H&E or after anti cleaved-Caspase3 immuno-staining as previously described [3]. For proliferation analysis, mice were injected with 0.25 ml of BrdU (Amersham) before culling and were taken either 2 hrs (day4) or 24 hrs (day5) after BrdU injection. Staining was performed as previously described [3]. The number of cells in *AhCre^+^Apc^fl/fl^* and *AhCre^+^Apc^fl/fl^Cited1^−^* hyperplastic area was scored using the position of the last BrdU positive cells in the hyperplastic area. For each analysis, 25 full crypts or areas were scored from at least 3 mice of each genotype and time point.

### In situ hybridization (ISH)

In situ hybridization of *Olfm4* and *Cited1* in the small intestine was performed for all genotypes using sections embedded in paraffin sectioned at 5 µm. *Olfm4* hybridization was performed as described in Gregorieff et al., 2005. [48]. *Cited1* hybridization was performed using a probe against the sequence deleted in the *Cited1^−^* allele designed by Advanced cell Diagnostics inc (ACD). RNAscope 2.0 FFPE Reagent Kit – Brown kit was used according manufacturer instructions. Negative control Probe-DapB was used together with a positive control probe Polr2a from the ACD manufacturer.

### Microarray data analysis

The DNA microarray were performed from three mice of each genotype using Mouse Genome 430 2.0 Affymetrix chips at Liverpool Microarray Facility according to the manufacturer's instructions. The Microarray data were analyzed using AffylmGUI (Affymetrix linear modeling Graphical User Interface; http://bioinf.wehi.edu.au/affylmGUI/#citation) [26]. The p values presented have been corrected for multiple testing using the BH method to control the false discovery rate. The B statistic is the log odds that the gene is differentially expressed and is adjusted for multiple testing using the assumption that 1% of genes are expected to be differentially expressed [26], [49]–[51]. Microarray data were deposited in MIAME format at www.ebi.ac.uk/arrayexpress/ (Accession Number: E-MEXP-3202)

QPCR protocols, routine methods and a description of the statistical analyses used are provided in **Protocol S1**. List of primers for Taqman RT-QPCR, Sybr green RT-QPCR, and Cited1 semi quantitative RT-PCR are provided in Figure S4.

### Analysis of signaling pathways

Ingenuity pathway analysis (IPA) software (www.ingenuity.com) was used to determine which signaling pathways were affected by the loss of *Cited1* in *AhCre^+^WT* or *AhCre^+^Apc^fl/fl^* mice. The comparative (*AhCre^+^WT* vs *AhCre^+^Cited1* ^−^ and *AhCre^+^Apc^fl/fl^* vs *AhCre^+^Apc^fl/fl^Cited1^−^*) data from the microarray analysis were filtered for a p value of less than 0.05 and imported into the IPA software. The significance of the association between the data set and the pathway was measured in 2 ways: by the ratio and by a p value. The ratio corresponds to the number of genes from our data set that map to the ingenuity pathway divided by the total number of genes that map to the Ingenuity canonical pathway. The p value is calculated by a right tailed Fischer's exact test. The p-value associated with a pathway is a measure of the likelihood that the association between a set of focus genes in your experiment and a pathway is due to random chance.

### β-catenin suspension bead array based assay

Analysis of biological function, localization, and posttranslational modification of the different forms of β-catenin were carried out as previously described [32]. Two additional assays were included in the analysis. Anti-dephospho S33/S37 and T41 (Cell Signalling Technologies) was used as an additional capture antibody to measure dephosphorylated β-catenin and GST-ICAT was employed as an additional bait protein to study free β-catenin.

## Supporting Information

Figure S1 RT-PCR for *Cited 1* expression in *Apc^Min/+^* mice. A: RT-PCR products showing up-regulation of *Cited1* in polyps (T) compared to normal intestinal tissue (N) in *Apc^Min/+^*. Note the absence of RT-PCR products in *Apc^Min/+^Cited1^−^* condition confirming the loss of *Cited1* expression. β-actin was used as internal positive control. B: RT-PCR of the Apc recombined cDNA in *AhCre^+^Apc^fl/fl^* and *AhCre^+^Apc^fl/fl^Cited1^−^*. The Apc non recombined cDNA (wild type Apc) gives a band of 383 bp whereas the Apc recombined cDNA (Apc Rec) gives a PCR product of 168 bp. C: Percentage tumour distribution varies in *Apc^Min/+^* mice compared to *Apc^Min/+^Cited1^−^* mice. The percentage tumour distribution was analysed by counting tumour burden in each of 5 cm sections along the length of the small intestine and representing this as a percentage of total tumour burden per section. Sections 1–2 (duodenum), 3–5 (jejunum) 6–8 (comparable to human Ileum). The colon was similarly divided into 1 cm sections. Section 1 corresponding to the rectum. Error bars represent standard errors. *p<0.05; statistical tests was done using Mann-Whitney U test.
(TIF)

Figure S2 Increased level of dephosphorylated-β-catenin and deregulated pathways in *AhCre^+^Apc^fl/fl^Cited1^−^* compared to *AhCre^+^Apc^fl/fl^*. A: Western blot analysis of the active form of β-catenin (92 kD) using dephospho-β-catenin (Non-phospho-β-Catenin Ser33/37/Thr41,Cell signalling) antibody in *AhCre^+^WT (WT)*, *AhCre^+^Cited1 (Cited1^−^)*, *AhCre^+^Apc^fl/fl^* (*Apc^fl/fl^*) and *AhCre^+^Apc^fl/fl^Cited1^−^(Apc^fl/fl^Cited1^−^*). There is a strong up-regulation of dephospho-β-catenin in *AhCre^+^Apc^fl/fl^* compared to *AhCre^+^WT* and *AhCre^+^Cited1^−^* and the level of dephospho-β-catenin is further elevated in *AhCre^+^Apc^fl/fl^Cited1^−^* compared to *AhCre^+^Apc^fl/fl^*. B: The histogram represents the densitometry analysis of the dephospho-β-catenin immumo-blot normalised to the internal control *β-actin*. *AhCre^+^Apc^fl/fl^Beta-cat^fl/fl^* (*Apc^fl/fl^Beta-cat^fl/fl^*) is used as a negative control. C: A: Fold change of target genes expression (other than Wnt) in the small intestinal epithelium of *AhCre^+^Apc^fl/fl^Cited1^−^* compared to *AhCre^+^Apc^fl/fl^* mice measured by QRT-PCR, *p<0.05 Mann Whitney U test. D: Microarray analysis showing up-regulation of Wnt target genes with Wnt Key players in *AhCre^+^WT* compared to *AhCre^+^Cited1^−^* and *AhCre^+^Apc^fl/fl^* compared to *AhCre^+^WT*: Fold changes (FC) are presented with correspondent P value and B value (B statistic is lod score).
(TIF)

Figure S3 *Cited1* deficiency does not modify the renal carcinoma phenotype induced after loss of *Apc* and activation of *K-ras^V12^*. Cre recombinase under the Cyp1A promoter has also been shown to be constitutively expressed in a proportion of cells in the renal epithelium [reference S1, in Protocd S1]. This drives loss of the Apc allele and the formation of dysplastic foci characterised by accumulation of nuclear β-catenin. Within 4 months, mice develop renal carcinoma [reference S1, in Protocd S1] which can be accelerated by an additional K-ras^V12^ oncogene [reference S2, in Protocd S1]. To study the role of Cited1 in renal cell carcinoma, mice *AhCre^+^Apc^fl/fl^ K-ras^V12^*, *AhCre^+^Apc^fl/fl^ K-ras-^V12^ Cited^−/+^* and *AhCre^+^Apc^fl/fl^ K-ras-^V12^Cited1^−^* were generated and maintained on an outbred background. All experiments were performed under the UK Home Office guidelines. *K-ras^V12^* allele was obtained and genotyped as previously described [reference S3, in Protocd S1]. Mice were sacrificed at ill health. We analysed expression of *Cited1* in the renal carcinomas of *AhCre^+^Apc^fl/fl^K-ras^V12^* compared to normal tissue. Histograms (A) are showing qRT-PCR delta CT values (left panel) and Fold change (right panel) for *Cited1* expression in the kidneys of *AhCre^+^WT*, *AhCre^+^Apc^fl/fl^ K-ras^V12^* (tumours) and *AhCre^+^Apc^fl/fl^K-rasv^12^Cited1^−^* (tumours) mice. There is a significant 19.48 fold increase in *Cited1* expression in *AhCre^+^Apc^fl/fl^K-ras^V12^* mice kidneys compared to *WT* mice (p = 0.0041 Mann-Whitney U test) and 1007 fold change difference between *AhCre^+^Apc^fl/fl^K-ras^V12^* and *AhCre^+^Apc^fl/fl^K-ras^V12^Cited1^−^* (p = 0.0071 Mann-Whitney U test). (B) We generated cohorts of *AhCre^+^Apc^fl/fl^K-ras^V12^*, *AhCre^+^Apc^fl/fl^K-ras^V12^Cited1^−^* and *AhCre^+^Apc^fl/fl^K-ras^V12^Cited1^−/+^* mice and monitored them for signs of illness. The Kaplan-Meier shows no significant difference in survival between *AhCre^+^Apc^fl/fl^K-ras^V12^* mice (n = 13) (dashed) versus *AhCre^+^Apc^fl/fl^K-ras^v12^Cited1^−^* (n = 4) (Bold solid line; p = 0.732, Log-Rank test) and between *AhCre^+^Apc^fl/fl^K-ras^v12^* versus *AhCre^+^Apc^fl/fl^K-ras^V12^Cited1^−/+^* (n = 5) (Thin solid line; p = 0.555 Log-Rank test). The median lifespan was 88 days in *AhCre^+^Apc^fl/fl^K-ras^V12^* (n = 13), 72 days in *AhCre^+^Apc^fl/fl^K-ras^V12^Cited1^−^* (n = 4) and 81 days in *AhCre^+^Apc^fl/fl^K-ras^V12^Cited1^−/+^* (*AhCre^+^Apc^fl/fl^K-ras^V12^ vs AhCre^+^Apc^fl/fl^K-ras^V12^Cited1^−^*, Log Rank p = 0.732; *AhCre^+^Apc^fl/fl^K-ras^V12^ vs AhCre^+^Apc^fl/fl^K-ras^V12^Cited1^−/+^* Log Rank p = 0.555). These data indicate that *Cited1* deficiency does not modify the survival of mice affected by renal carcinoma and that *Cited1* does not play a role in renal carcinoma induced by a loss of Apc.
(TIF)

Figure S4 Primers Table. Tables listing the primers and probes used for Taqman quantitative PCR in human (A) and in mice (B); Sybr Green quantitative real time PCR in mice (C) and semi- quantitative PCR in mice (D).
(TIF)

Figure S5 Cited1 deficiency further represses the number of differentiated cell types after Apc loss. To determine if deficiency of *Cited1* modifies cell differentiation along the crypt-villus axis, we analysed the presence and location of several secretory cell types using markers of cell lineage in mice intestinal epithelium of all 4 genotypes: goblet cells (A–B) (Alcian Blue staining and counting), enteroendocrine cells (C–D) (Grimelius staining and counting) and paneth cell (E–F) (Lysozyme staining and counting). (G) Paneth cells position was analysed. Goblet cells and enteroendocrine cells were scored in 25 crypts (or hyperplastic areas)-villus (n = 6/genotype) (All statistical analysis were done using the Mann-Whitney U test and NS = Non significant). We found that the number of goblet cells and enteroendocrine cells were not significantly different in *AhCre^+^WT* mice compared to *AhCre^+^Cited1^−^* mice (Goblet cells = *AhCre^+^WT*: 9.8 cells/crypt-villus vs *AhCre^+^Cited1^−^*: 11.4 cells/crypt-villus, p = 0.1312, ; Enteroendocrine cells = *AhCre^+^WT*: 1.56 cells/crypt-villus *vs AhCre^+^Cited1^−^*: 1.61 cells/crypt-villus, p = 0.5) (B,D). However, the number of goblet cells and enteroendocrine cells per hyperplastic area-villus were both significantly reduced in *AhCre^+^Apc^fl/fl^Cited1^−^* mice compared to *AhCre^+^Apc^fl/fl^* mice (Goblet cells = *AhCre^+^Apc^fl/fl^* : 5.26 cells/area-villus *vs AhCre^+^Apc^fl/fl^Cited1^−^*: 3.71 cells/area-villus, p = 0.0463, Enteroendocrine cells = *AhCre^+^Apc^fl/fl^*: 1.04 cells/area-villus vs *AhCre^+^Apc^fl/fl^Cited1^−^*: 0.73 cells/area-villus, p = 0.0125) (B,D). The position and the number of paneth cells are not modified in *AhCre^+^Cited1^−^* mice compared to *AhCre^+^WT* mice (E–G). After loss of *Apc*, paneth cells lose their position at the bottom of the crypt and are mislocalised along the crypt-villus axis (G). We observe a change in position of the paneth cells in the hyperplastic areas of the *AhCre^+^Apc^fl/fl^Cited1^−^* compared to *AhCre^+^Apc^fl/fl^* (E–G). Statistical test was done using Kolmogorov–Smirnov test (G). Inset panels show magnifications of intestine of corresponding zone. Taken altogether, these data suggest that loss of *Cited1* does not perturb the pattern of differentiation when *Apc* is present. However in the absence of *Apc*, *Cited1* deficiency appears to accentuate the phenotype, with a more extreme reduction in the number of differentiated cell types.
(TIF)

Protocol S1 Statistical Analysis, Quantitative real-time PCR on mice samples, *Cited1* status by Semi-quantitative PCR, First strand cDNA synthesis and taqman quantitative qPCR on human samples, Western Blot analysis, RNA and protein isolation from mice small intestine, Histology and Immunohistochemistry and Supplementary references are provided as supplementary informations. Primers list for Taqman RT-QPCR, Sybr green RT-QPCR and semi quantitative RT-PCR are listed in Figure S4.
(DOC)
