# Supplementary figures and images for: Cited1 Deficiency Suppresses Intestinal Tumorigenesis
Source: PLoS Genet. 2013 Aug 1;9(8):e1003638. doi: 10.1371/journal.pgen.1003638 (PMC3731217; doi:10.1371/journal.pgen.1003638)

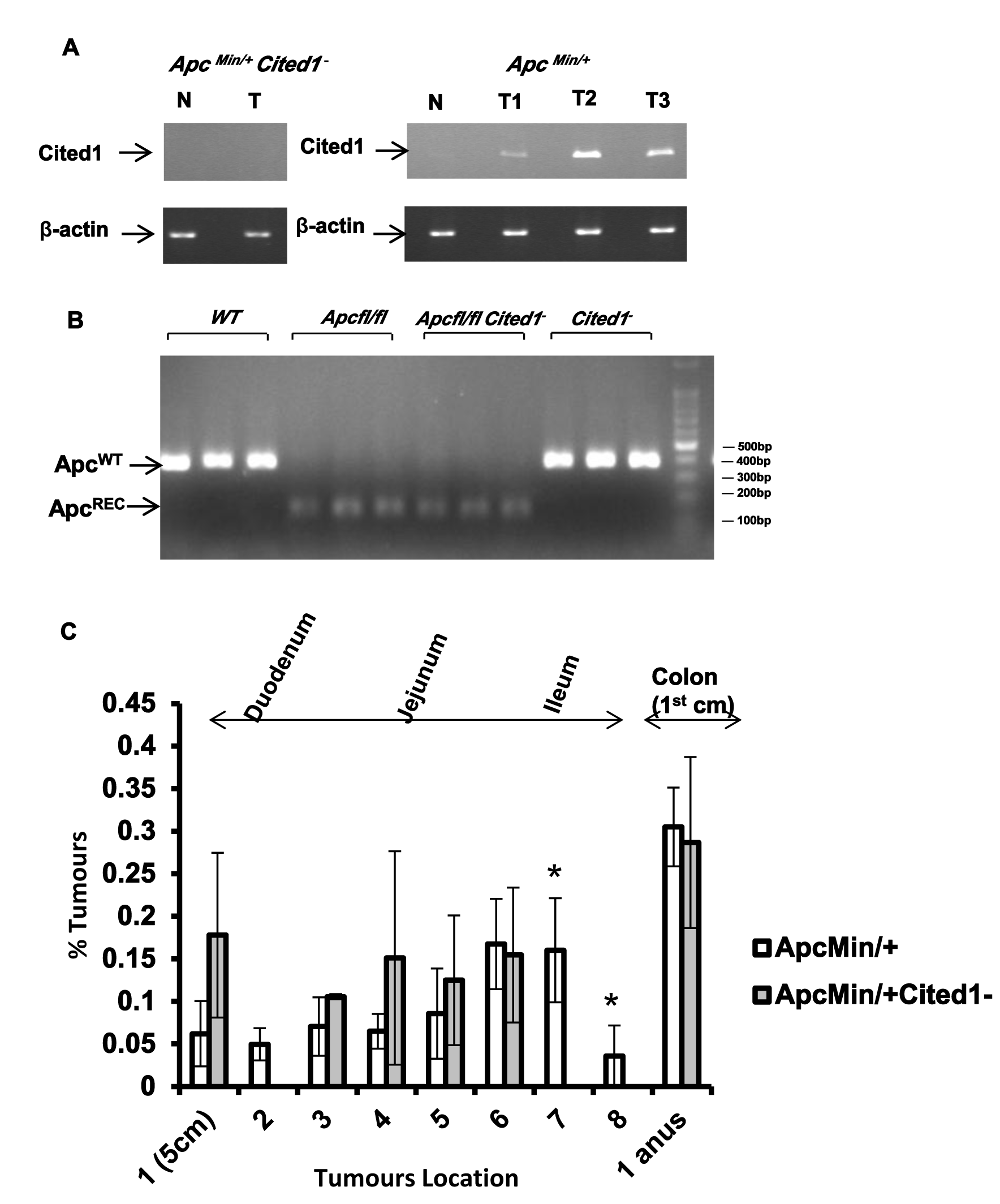

Supplement: Figure S1 — RT-PCR for Cited 1 expression in ApcMin/+ mice. A: RT-PCR products showing up-regulation of Cited1 in polyps (T) compared to normal intestinal tissue (N) in ApcMin/+. Note the absence of RT-PCR products in ApcMin/+Cited1− condition confirming the loss of Cited1 expression. β-actin was used as internal positive control. B: RT-PCR of the Apc recombined cDNA in AhCre+Apcfl/fl and AhCre+Apcfl/flCited1−. The Apc non recombined cDNA (wild type Apc) gives a band of 383 bp whereas the Apc recombined cDNA (Apc Rec) gives a PCR product of 168 bp. C: Percentage tumour distribution varies in ApcMin/+ mice compared to ApcMin/+Cited1− mice. The percentage tumour distribution was analysed by counting tumour burden in each of 5 cm sections along the length of the small intestine and representing this as a percentage of total tumour burden per section. Sections 1–2 (duodenum), 3–5 (jejunum) 6–8 (comparable to human Ileum). The colon was similarly divided into 1 cm sections. Section 1 corresponding to the rectum. Error bars represent standard errors. *p<0.05; statistical tests was done using Mann-Whitney U test. (TIF) [file pgen.1003638.s001.tif]

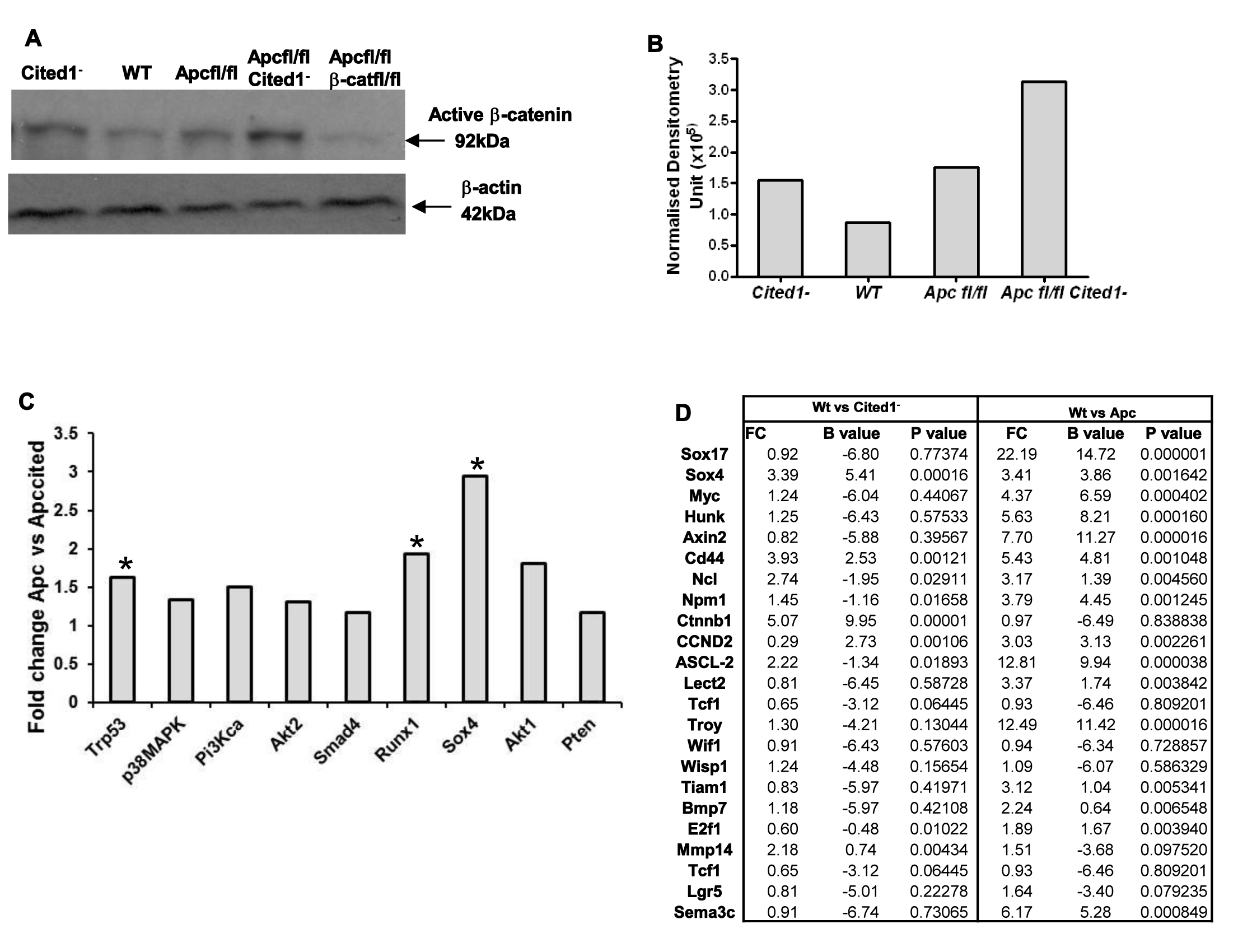

Supplement: Figure S2 — Increased level of dephosphorylated-β-catenin and deregulated pathways in AhCre+Apcfl/flCited1− compared to AhCre+Apcfl/fl. A: Western blot analysis of the active form of β-catenin (92 kD) using dephospho-β-catenin (Non-phospho-β-Catenin Ser33/37/Thr41,Cell signalling) antibody in AhCre+WT (WT), AhCre+Cited1 (Cited1−), AhCre+Apcfl/fl (Apcfl/fl) and AhCre+Apcfl/flCited1−(Apcfl/flCited1−). There is a strong up-regulation of dephospho-β-catenin in AhCre+Apcfl/fl compared to AhCre+WT and AhCre+Cited1− and the level of dephospho-β-catenin is further elevated in AhCre+Apcfl/flCited1− compared to AhCre+Apcfl/fl. B: The histogram represents the densitometry analysis of the dephospho-β-catenin immumo-blot normalised to the internal control β-actin. AhCre+Apcfl/flBeta-catfl/fl (Apcfl/flBeta-catfl/fl) is used as a negative control. C: A: Fold change of target genes expression (other than Wnt) in the small intestinal epithelium of AhCre+Apcfl/flCited1− compared to AhCre+Apcfl/fl mice measured by QRT-PCR, *p<0.05 Mann Whitney U test. D: Microarray analysis showing up-regulation of Wnt target genes with Wnt Key players in AhCre+WT compared to AhCre+Cited1− and AhCre+Apcfl/fl compared to AhCre+WT: Fold changes (FC) are presented with correspondent P value and B value (B statistic is lod score). (TIF) [file pgen.1003638.s002.tif]

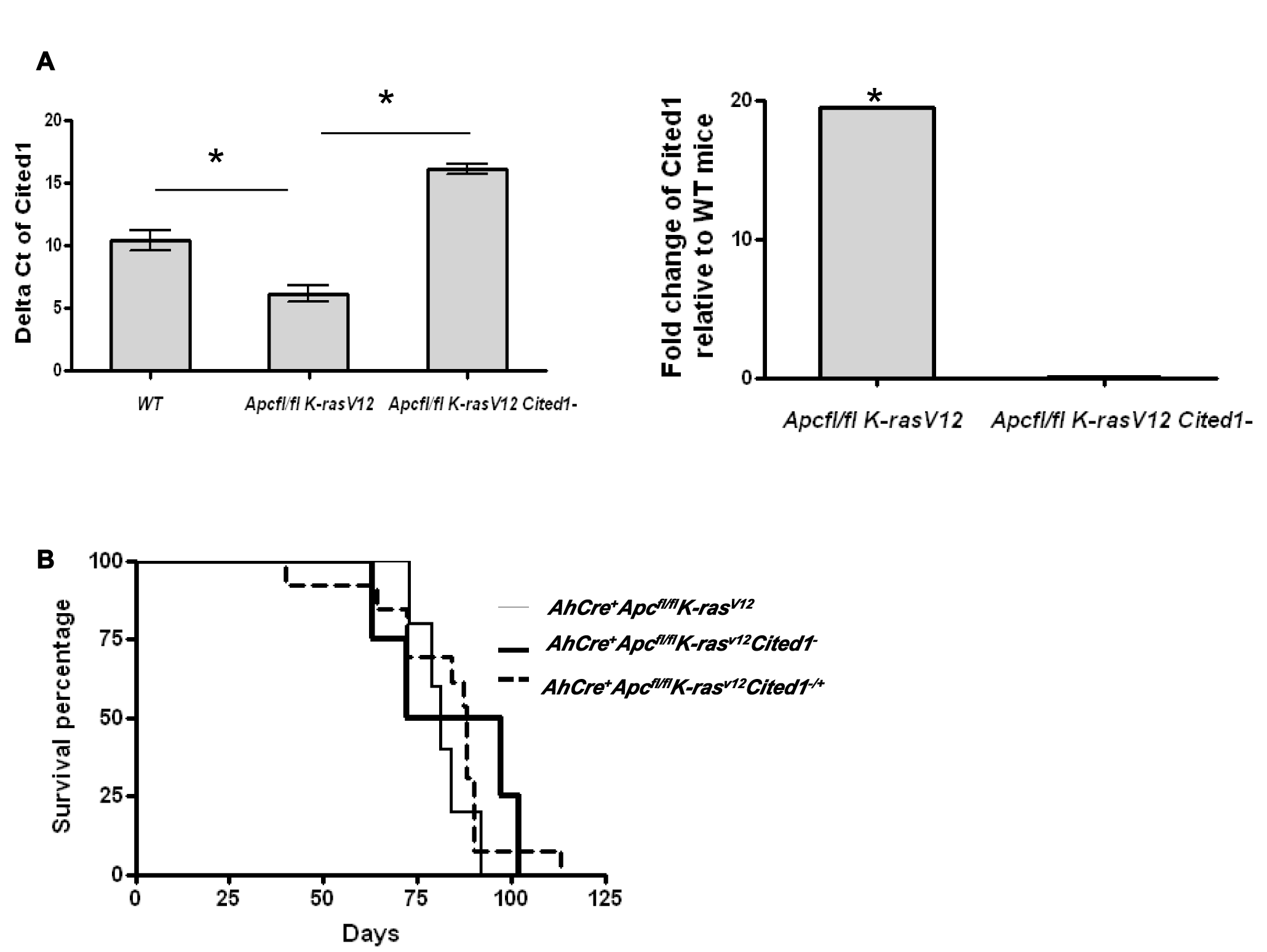

Supplement: Figure S3 — Cited1 deficiency does not modify the renal carcinoma phenotype induced after loss of Apc and activation of K-rasV12. Cre recombinase under the Cyp1A promoter has also been shown to be constitutively expressed in a proportion of cells in the renal epithelium [reference S1, in Protocd S1]. This drives loss of the Apc allele and the formation of dysplastic foci characterised by accumulation of nuclear β-catenin. Within 4 months, mice develop renal carcinoma [reference S1, in Protocd S1] which can be accelerated by an additional K-rasV12 oncogene [reference S2, in Protocd S1]. To study the role of Cited1 in renal cell carcinoma, mice AhCre+Apcfl/fl K-rasV12, AhCre+Apcfl/fl K-ras-V12 Cited−/+ and AhCre+Apcfl/fl K-ras-V12Cited1− were generated and maintained on an outbred background. All experiments were performed under the UK Home Office guidelines. K-rasV12 allele was obtained and genotyped as previously described [reference S3, in Protocd S1]. Mice were sacrificed at ill health. We analysed expression of Cited1 in the renal carcinomas of AhCre+Apcfl/flK-rasV12 compared to normal tissue. Histograms (A) are showing qRT-PCR delta CT values (left panel) and Fold change (right panel) for Cited1 expression in the kidneys of AhCre+WT, AhCre+Apcfl/fl K-rasV12 (tumours) and AhCre+Apcfl/flK-rasv12Cited1− (tumours) mice. There is a significant 19.48 fold increase in Cited1 expression in AhCre+Apcfl/flK-rasV12 mice kidneys compared to WT mice (p = 0.0041 Mann-Whitney U test) and 1007 fold change difference between AhCre+Apcfl/flK-rasV12 and AhCre+Apcfl/flK-rasV12Cited1− (p = 0.0071 Mann-Whitney U test). (B) We generated cohorts of AhCre+Apcfl/flK-rasV12, AhCre+Apcfl/flK-rasV12Cited1− and AhCre+Apcfl/flK-rasV12Cited1−/+ mice and monitored them for signs of illness. The Kaplan-Meier shows no significant difference in survival between AhCre+Apcfl/flK-rasV12 mice (n = 13) (dashed) versus AhCre+Apcfl/flK-rasv12Cited1− (n = 4) (Bold solid line; p = 0.732, Log-Rank test) and between AhC [file pgen.1003638.s003.tif]

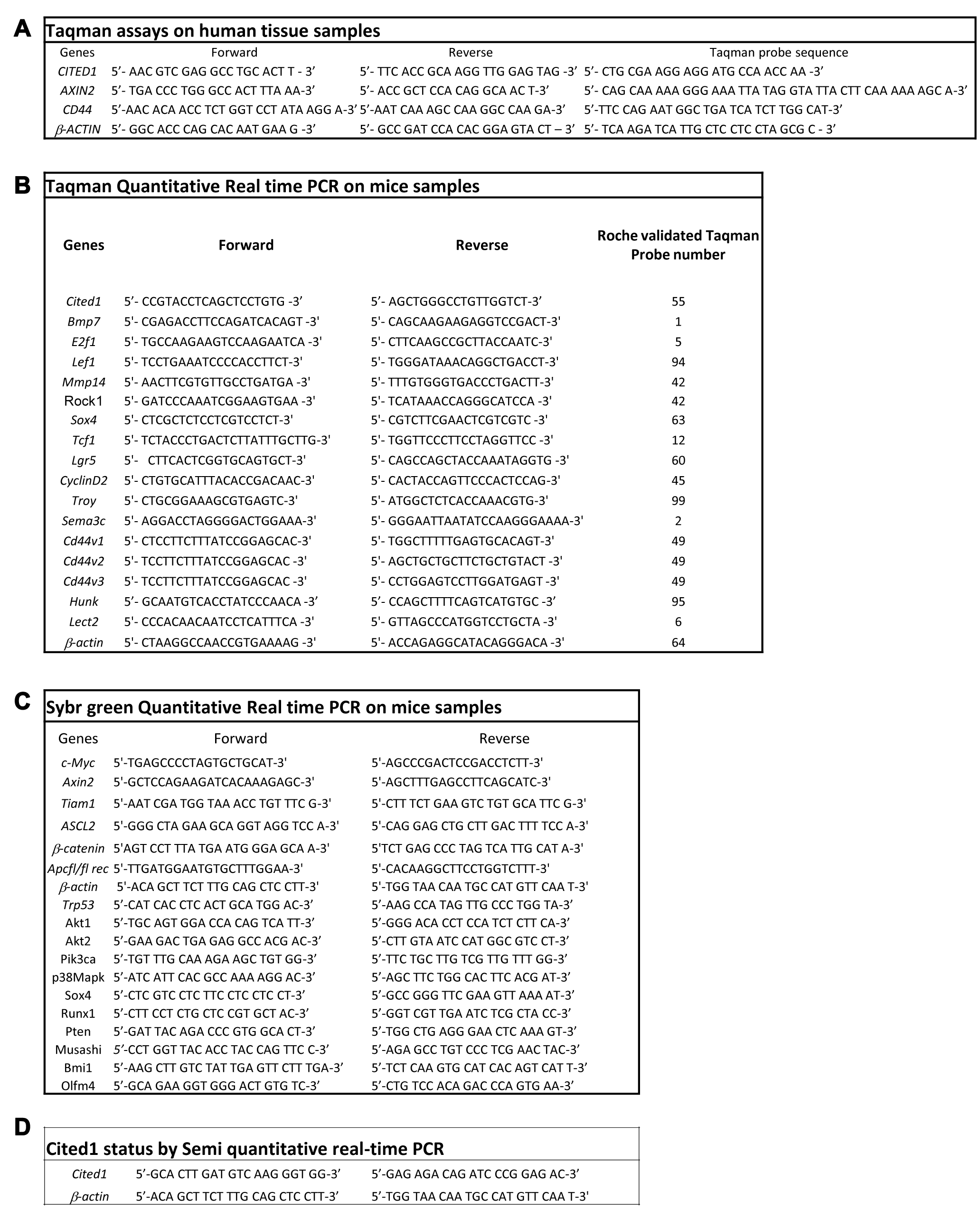

Supplement: Figure S4 — Primers Table. Tables listing the primers and probes used for Taqman quantitative PCR in human (A) and in mice (B); Sybr Green quantitative real time PCR in mice (C) and semi- quantitative PCR in mice (D). (TIF) [file pgen.1003638.s004.tif]

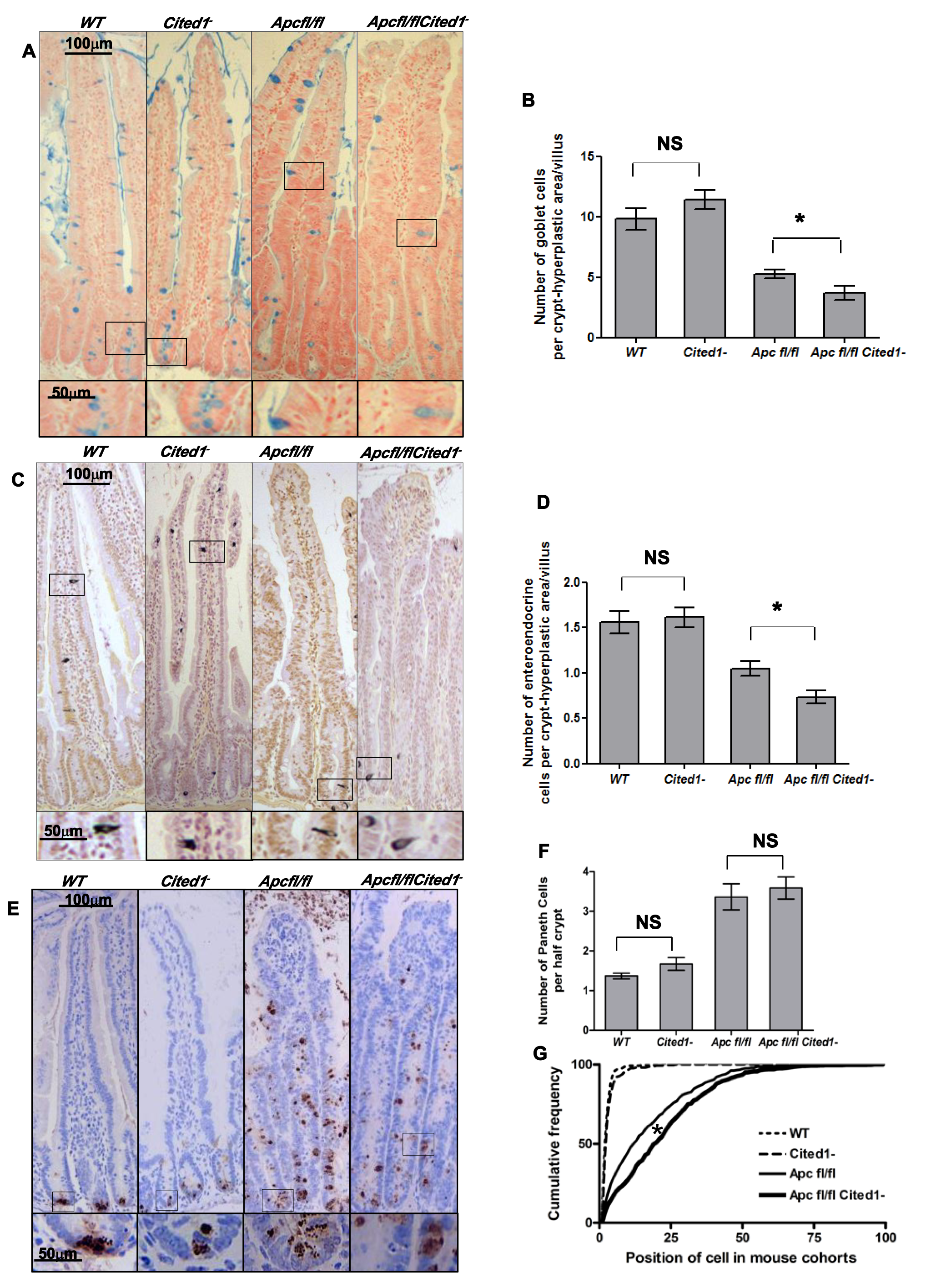

Supplement: Figure S5 — Cited1 deficiency further represses the number of differentiated cell types after Apc loss. To determine if deficiency of Cited1 modifies cell differentiation along the crypt-villus axis, we analysed the presence and location of several secretory cell types using markers of cell lineage in mice intestinal epithelium of all 4 genotypes: goblet cells (A–B) (Alcian Blue staining and counting), enteroendocrine cells (C–D) (Grimelius staining and counting) and paneth cell (E–F) (Lysozyme staining and counting). (G) Paneth cells position was analysed. Goblet cells and enteroendocrine cells were scored in 25 crypts (or hyperplastic areas)-villus (n = 6/genotype) (All statistical analysis were done using the Mann-Whitney U test and NS = Non significant). We found that the number of goblet cells and enteroendocrine cells were not significantly different in AhCre+WT mice compared to AhCre+Cited1− mice (Goblet cells = AhCre+WT: 9.8 cells/crypt-villus vs AhCre+Cited1−: 11.4 cells/crypt-villus, p = 0.1312, ; Enteroendocrine cells = AhCre+WT: 1.56 cells/crypt-villus vs AhCre+Cited1−: 1.61 cells/crypt-villus, p = 0.5) (B,D). However, the number of goblet cells and enteroendocrine cells per hyperplastic area-villus were both significantly reduced in AhCre+Apcfl/flCited1− mice compared to AhCre+Apcfl/fl mice (Goblet cells = AhCre+Apcfl/fl : 5.26 cells/area-villus vs AhCre+Apcfl/flCited1−: 3.71 cells/area-villus, p = 0.0463, Enteroendocrine cells = AhCre+Apcfl/fl: 1.04 cells/area-villus vs AhCre+Apcfl/flCited1−: 0.73 cells/area-villus, p = 0.0125) (B,D). The position and the number of paneth cells are not modified in AhCre+Cited1− mice compared to AhCre+WT mice (E–G). After loss of Apc, paneth cells lose their position at the bottom of the crypt and are mislocalised along the crypt-villus axis (G). We observe a change in position of the paneth cells in the hyperplastic areas of the AhCre+Apcfl/flCited1− compared to AhCre+Apcfl/fl (E–G). Statistical test was done using Kolmogorov–Smir [file pgen.1003638.s005.tif]
