# Supplementary material for: Cited1 Deficiency Suppresses Intestinal Tumorigenesis
Source: PLoS Genet. 2013 Aug 1;9(8):e1003638. doi: 10.1371/journal.pgen.1003638 (PMC3731217; doi:10.1371/journal.pgen.1003638)
Supplement: Protocol S1 — Statistical Analysis, Quantitative real-time PCR on mice samples, Cited1 status by Semi-quantitative PCR, First strand cDNA synthesis and taqman quantitative qPCR on human samples, Western Blot analysis, RNA and protein isolation from mice small intestine, Histology and Immunohistochemistry and Supplementary references are provided as supplementary informations. Primers list for Taqman RT-QPCR, Sybr green RT-QPCR and semi quantitative RT-PCR are listed in Figure S4. (DOC) [file pgen.1003638.s006.doc]

***Cited1* deficiency suppresses intestinal tumorigenesis.**

Valérie Méniel^1^*, Fei Song^2a,b^*, Toby Phesse^3^*, Madeleine Young^1^, Oliver Poetz^4^, Lee Parry^1^, John R Jenkins^2^, , Geraint T Williams^5^, Sally L Dunwoodie^6a,b^, Alastair Watson^7#^, Alan R Clarke^1^.

Supplementary Materials:

Supplementary methods.

## Statistical Analysis Comparison between the median of a single column of data against a hypothetical median was performed on relative fold changes of candidate gene expressions in human colorectal tumours using Wilcoxon Signed Rank Test (GraphPad PRISM for Windows v4). In the *in vivo* *Cited1* knockout studies, comparison of medians of delta Ct data sets and tumour burden between mouse cohorts were performed using Mann-Whitney U test (MiniTab for Windows v15). Comparison of median lifespan of murine cohorts was performed on survival percentage using Kaplan-Meier method and analysed by Log-Rank test (MiniTab for Windows v15). Comparison of maximum vertical distance of cell proliferation and cell migration was performed on cumulative frequency between mouse cohorts using the Kolmogorov-Smirnov test [reference S4]. P values, where applicable, were assigned to indicate statistical significance (P<0.05 = * ; P<0.01 = **). Error bars indicate standard error of the mean (SEM).

**Quantitative real-time PCR** **on mice samples**

First strand cDNA were synthesized using Verso^TM^ cDNA Kit (Thermo Scientific) and anchored oligo-dT primers (Thermo Scientific) and quantitative real-time PCR were performed as described above using Roche pre-validated Taqman assays in order to assess the expression of *Cited1*, *Bmp7, E2f1, Lef1, Mmp14, Rock1, Sox4, Tcf1, Lgr5, CyclinD2, Troy, Sema3c, Cd44, Hunk* and *Lect2. β-actin* was used as control reference gene. The primer and probe sequences are listed in Primers Table (Figure S4): The expression of *c-Myc*, *Axin2*, *Tiam1*, *Ascl2*, *β-catenin*, recombined *Apc^fl/fl^*, *Cited1*, *P53, p38Mapk, Pi3Kca, Akt1, Akt2, Smad4, Runx1, Sox4, Pten, β-actin* were also analysed with purified RNA treated as previously described [reference S5]. Reactions were run on a PTC-200 thermal cycler and a Chromo4 continuous fluorescence detector (both from MJ Research), the analysis was performed using Opticon Monitor3 software (MJ Research). The thermal amplification conditions are: 95°C for 15 min; 95°C 30 sec, 60°C for 30 sec, 72°C for 30 sec 40 cycles.

***Cited1* status by Semi-quantitative PCR**

*Cited1* expression in the polyps and adjacent normal tissue (3cm of the small intestine located 7cm from the stomach) of *Apc^Min/+^* and *Apc^Min/+^ Cited1^-^* mice was analysed by Semi-quantitative real-time PCR. RNA was isolated using Trizol reagent and the cDNA was done as previously described [reference S5]. *Cited1* primers used are listed in Primers Table in Figure S4 [reference S6]. *β-actin* expression was used to standardize the concentration of cDNA samples. Semi-quantitative PCR was performed as follows: *Cited1*: 98 ^o^C 1 min; 98 ^o^C 10 sec, 60 ^o^C 30 sec, 72 ^o^C 30 secs for 28 cycles; 72 ^o^C for 5 min using Phusion Taq polymerase. For *β-actin*, 94 ^o^C 2.5 min; 94 ^o^C 30 sec, 60 ^o^C 30 sec, 72 ^o^C 1 min for 30 cycles; 72 ^o^C 5 min using GoTaq (Promega).

**First strand cDNA synthesis and taqman quantitative qPCR on human samples**

Quantified total RNA of human colorectal cancer tissue samples were used to synthesize the first strand cDNA using Verso^TM^ cDNA Kit (Thermo Scientific) and anchored oligo-dT primers (Thermo Scientific).

Taqman assays for *CITED1*, *AXIN2*, *CD44* and *β-ACTIN* were designed by using Primer Express program (Applied Biosystems) and the sequences confirmed as specific by NCBI Nucleotide-BLAST search. The synthesised primers and probes (Eurogentec) were optimised as described by Applied Biosystems. Taqman assays of patient samples were performed in triplicate using qPCR Core Kit (Eurogentec) on ABI PRISM 7700 Sequence Detection System with Sequence Detection System software (version 1.7 alias) (Applied Biosystems). *β-ACTIN* was used as the loading control. The primer and probe sequences were as listed in primers Table (Figure S4). The thermal amplification conditions are as follows: 50°C 2 min; 95°C 10 min; 95°C 15 sec, 60°C 1 min for 40 cycles. The gene transcript levels were presented as ΔΔCt relative to control and the final relative fold changes were calculated as 2 to the power of ΔΔCt.

**Western Blot analysis**

30 µg of proteins were separated as previously described [reference S5]. For the active form of β-catenin we used two different antibodies: Non-phospho-β-Catenin (ser33/37/Thr41) antibody (1/3000, Cell signalling) and the Anti-Active-β-Catenin (anti-ABC), clone 8E7 which recognizes the active form of β-catenin, dephosphorylated on Ser37 or Thr41 (1/2000, Millipore), and to detect Total β-catenin we used an antibody raised against the Amino-terminal Antigen (NEB 9581;1/3000) . For the internal control, we used a mouse antibody to β-actin (1:5,000; Sigma) or α-tubulin antibody (Abcam at 1/2000). Densitometry analysis was performed using Quantiscan software.

**RNA and protein isolation from mice small intestine**

RNA and protein from mice small intestine were extracted from enriched epithelial cells as described [reference S5] and using a Weiser chelating solution [reference S7]. Epithelial cell pellets were homogenised in Trizol reagent (InVitrogen) and RNA extracted as previously described [reference S5]. The mouse RNA samples were cleaned up using RNeasy Mini Kit (Qiagen) prior to the DNA microarray. Protein was extracted as previously described [reference S5].

**Histology and Immunohistochemistry.**

Intestinal tissue was fixed in 10% neutral buffered formalin or in Methacarn. Tissue sections (5 µm) were either stained with H&E for histological analysis or used for imunohistochemistry. Goblet cells were stained following the Alcian Blue protocols (<http://www.nottingham.ac.uk/pathology/protocols.html>) and enteroendocrine cells as previously described [3]. Paneth cells were stained using an anti-lysozyme antibody (A0099, Dako) after an antigen retrieval step with a proteolytic enzyme solution (S3007 Dako). Immunohistochemistry for β-catenin was performed with 1:50 anti-β-catenin primary antibody (BD C19220 transduction Laboratories) as described in [3].

**Supplementary references.**

S1. Sansom OJ, Griffiths DF, Reed KR, [Winton DJ](http://www.ncbi.nlm.nih.gov/pubmed?term="Winton DJ"%5BAuthor%5D), [Clarke AR](http://www.ncbi.nlm.nih.gov/pubmed?term="Clarke AR"%5BAuthor%5D). (2005) Apc deficiency predisposes to renal carcinoma in the mouse. Oncogene 24:8205-8210.

S2. Sansom OJ, Meniel V, Wilkins JA, [Cole AM](http://www.ncbi.nlm.nih.gov/pubmed?term="Cole AM"%5BAuthor%5D), [Oien KA](http://www.ncbi.nlm.nih.gov/pubmed?term="Oien KA"%5BAuthor%5D), et al. (2006) Loss of Apc allows phenotypic manifestation of the transforming properties of an endogenous K-ras oncogene in vivo. Proc Natl Acad Sci USA 103:14122-14127.

S3. Guerra C, Mijimolle N, Dhawahir A, [Dubus P](http://www.ncbi.nlm.nih.gov/pubmed?term="Dubus P"%5BAuthor%5D), [Barradas M](http://www.ncbi.nlm.nih.gov/pubmed?term="Barradas M"%5BAuthor%5D), et al. (2003)Tumor induction by an endogenous K-ras oncogene is highly dependent on cellular context. Cancer Cell 4:111-120.

S4. Sokal RR, and Rohl FJ. (1981) The principles and practice in biological research.; Second edition; W.H. Freeman and Company, San Francisco

S5. Marsh V, Winton DJ, Williams GT, Dubois N, Trumpp A, et al. (2008) Epithelial Pten is dispensable for intestinal homeostasis but suppresses adenoma development and progression after Apc mutation. Nat Genet 40:1436-1444.

S6. Boyle S, Shioda T, Perantoni AO, de Caestecker M (2007) Cited1 and Cited2 are differentially expressed in the developing kidney but are not required for nephrogenesis. Dev Dyn 236:2321-2330.

S7. Flint N, Cove FL, and Evans GS (1991) A low-temperature method for the isolation of small-intestinal epithelium along the crypt-villus axis. Biochem J *280 ( Pt 2)*; 331-334.
